# A *Drosophila* tumor model identifies a conserved Upd–JAK/STAT–Akh signaling axis associated with metabolic changes in cancer cachexia

**DOI:** 10.1242/dmm.052659

**Published:** 2026-06-16

**Authors:** Kewei Yu, Gurpreet S. Moroak, Esther M. Verheyen

**Affiliations:** Department of Molecular Biology and Biochemistry, Centre for Cell Biology, Development and Disease, Simon Fraser University, Burnaby, BC V5A 1S6, Canada

**Keywords:** Tumor, Cancer cachexia, Hipk, Sik3, Organ wasting, *Drosophila*, JAK/STAT, Unpaired, Brummer, Akh, Glucagon

## Abstract

Cancer-associated cachexia is a systemic wasting syndrome with no effective therapies, and it results in millions of deaths annually. Here, we established a *Drosophila* model of cancer cachexia using overexpression of Hipk and constitutively active Sik3 in larval epithelial tissue. Tumor-bearing larvae had significant muscle and fat body wasting, together with elevated carbohydrates and lipolysis. Mechanistically, tumors secrete Unpaired (Upd) ligands that activate JAK/STAT signaling in corpora cardiaca cells, inducing the expression of glucagon-like hormone Adipokinetic hormone (Akh). Elevated Akh, together with the lipase Brummer (Bmm), drives this systemic metabolic reprogramming and tissue catabolism. In conclusion, this study identifies a conserved tumor–host Upd–JAK/STAT–Akh signaling axis that contributes to organ wasting.

## Teaser

**Summary:** A *Drosophila* tumor model drives cancer cachexia and distant organ wasting through a conserved tumor–host signaling axis resulting in metabolic reprogramming.

## INTRODUCTION

Cancer-associated cachexia is a systemic paraneoplastic syndrome characterized by substantial weight loss due to loss of skeletal and adipose tissue, and it is estimated to cause an annual death rate of 2 million people worldwide (Baracos et al., 2018; Farkas et al., 2013; Fearon et al., 2011; Ferrer et al., 2023). These alterations in distant organs away from the site of the tumor are a result of the tumor altering the systemic metabolism of the host (Fearon et al., 2011; Porporato, 2016; Swanton et al., 2024). Thus far, there is no US Food and Drug Administration-approved drug therapy that can treat or reduce the progression of cancer cachexia (Baracos et al., 2018). This highlights the need to use model organisms to investigate the underlying mechanisms of tumor–host interactions resulting in cancer cachexia to facilitate potential new therapies targeting factors promoting cancer cachexia.

*Drosophila* is a well-established model for investigating cancer, with highly conserved genes and cell signaling pathways, as well as physiological conservation of organ functions (Bilder et al., 2021). In the recent decade, *Drosophila* tumor model studies have elucidated numerous cachexic factors and underlying signaling pathways mediating systemic organ wasting (Liu et al., 2022). For example, cachexic factors such as Unpaired (Upd) ligands (homologs of mammalian interleukins), ImpL2 [homolog of insulin-like growth factor-binding protein (IGFBP)], PDGF- and VEGF-related factor 1 (Pvf1; VEGF homolog) and Gbb [bone morphogenetic protein (BMP) homolog] have been found to induce host organ wasting coupled with abnormal metabolite levels in several different *Drosophila* adult and larval tumor models (Ding et al., 2021; Figueroa-Clarevega and Bilder, 2015; Kwon et al., 2015; Lodge et al., 2021; Song et al., 2019). Studies of patients with cancer cachexia have found that serum IGFBP2 is a biomarker for muscle wasting in patients with pancreatic ductal adenocarcinoma (Dong et al., 2021), and interleukin-6 (IL6) has been found to promote cancer cachexia in multiple types of human cancer and mouse models of cancer cachexia (Agca and Kir, 2024). These parallels show that *Drosophila* is a powerful model for understanding potential mechanisms of cancer cachexia.

Homeodomain-interacting protein kinases (Hipks) regulate cell proliferation, apoptosis and tissue development (Blaquiere and Verheyen, 2017). Previously, our laboratory established a larval tumor model based on overexpression of Hipk in *Drosophila* epithelial tissue. In this model, we observed hallmarks of cancer such as neoplasia, epithelial-to-mesenchymal transition (EMT) and increased aerobic glycolysis (Blaquiere et al., 2018; Wong et al., 2019). More recently, we described a tumor synergy between Hipk and salt-inducible kinases (Siks) 2 (Sik2) and 3 (Sik3) (Yu et al., 2023). Siks are serine/threonine kinases in the AMPK family (Wein et al., 2018). Humans possess three SIKs, whereas the fly genome encodes Sik2 and Sik3 (Choi et al., 2011). Siks can integrate conserved signaling pathways such as Notch and Hippo to promote tumor growth in *Drosophila* (Şahin et al., 2020; Wehr et al., 2013). Co-expression of constitutively active forms of Sik2 or Sik3 (Sik3-CA) with Hipk caused significant tissue hyperplasia and tissue distortion, accompanied by elevated levels of dMyc (also known as Myc), Armadillo (homolog of β-catenin) and the Yorkie target gene *expanded*, indicating that both Sik2 and Sik3 can synergize with Hipk to promote tumorous phenotypes (Yu et al., 2023). The human homolog of *dMyc*, *MYC*, is a well-characterized proto-oncogene and is estimated to have elevated or deregulated expression in up to 70% of human cancers (Dang, 2012). Wnt/β-catenin and the Hippo signaling pathways are also both often deregulated in human cancers (Harvey et al., 2013; Zhang and Wang, 2020). These findings indicate that Hipk and Siks are able to promote tumorigenesis through these conserved signaling pathways and through the proto-oncogene *dMyc*.

In the current study, we establish the ‘Hipk+Sik’ *Drosophila* tumor model as a cancer cachexia model by showing that hyperplasia and neoplasia induced by overexpression of Hipk and Sik3-CA in larval epithelial tissue causes muscle and fat wasting away from the site of the tumor. We characterized the aberrant metabolism in these tumor-bearing larvae and observed increased carbohydrates and lipolysis [breakdown of triacylglycerols (TAGs)]. We determined that this metabolic reprogramming is induced by both Adipokinetic hormone (Akh; functional homolog of mammalian glucagon) and Brummer [Bmm; the *Drosophila* homolog of mammalian adipose triglyceride lipase (ATGL)]. Finally, we show that Upd ligands secreted from the tumor tissue activate JAK/STAT signaling in corpora cardiaca (CC) cells to induce Akh expression. Therefore, we propose a *Drosophila* cancer cachexia model wherein tumors secrete Upd ligands to induce Akh in the distant organ CC cells, and Akh and Bmm converge to contribute to the aberrant metabolism, contributing to distant organ wasting.

## RESULTS

### *dpp>Hipk+Sik3-CA* induces functional changes in muscle and muscle wasting in *Drosophila* larvae

**Fig. 1.**
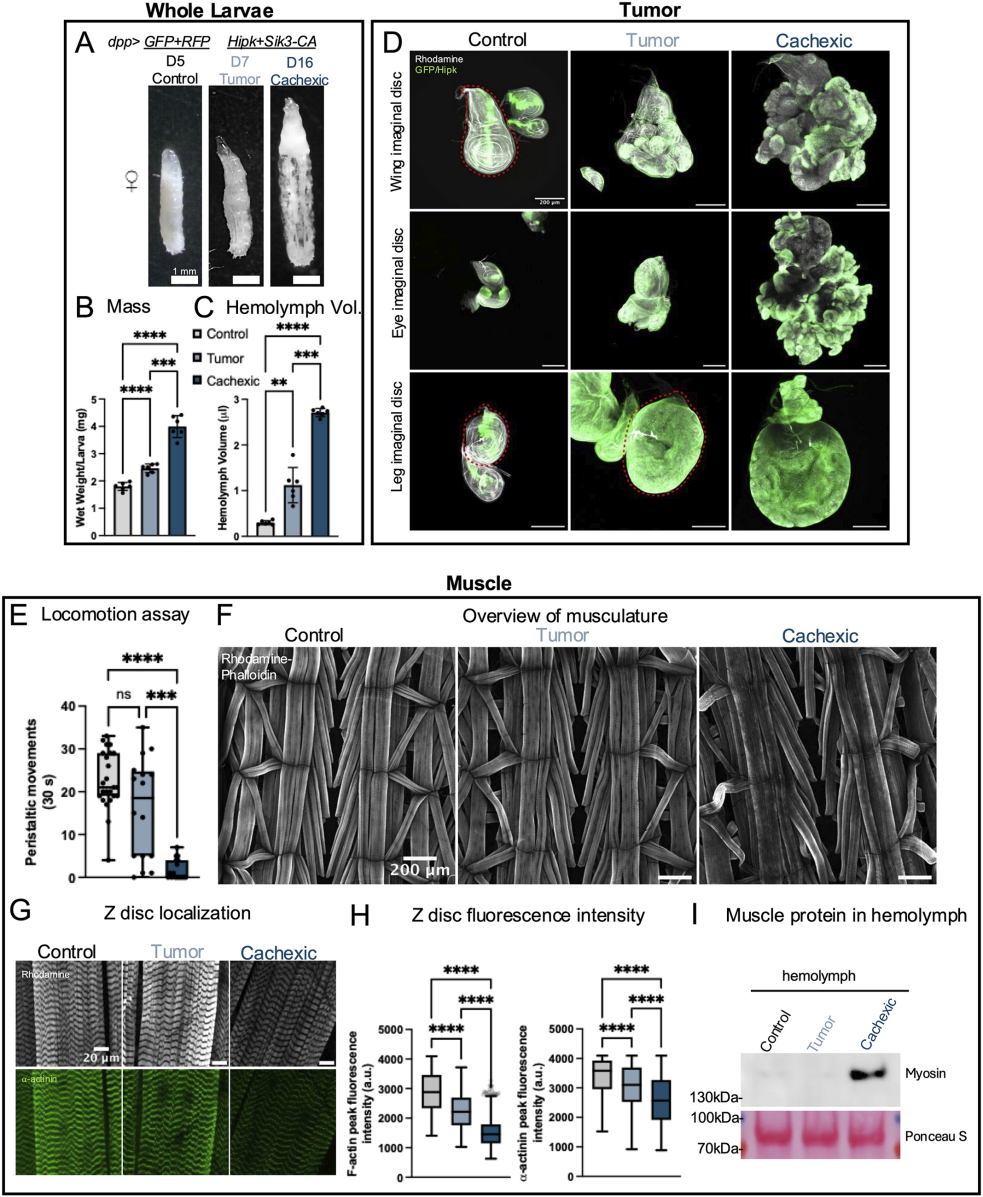
***dpp>Hipk+Sik3-CA* induces functional changes in muscle and muscle wasting in *Drosophila* larvae.** (A) Representative images of control, tumor-bearing and cachexic larvae (*n*=9, 13 and 15, respectively). D, day. (B) Wet weight of larvae with the indicated genotypes (*n*=5/data point) ***P=0.0001; ****P<0.0001 (Brown–Forsythe and Welch's one-way ANOVA test with Dunnett's T3 multiple comparisons test). (C) Hemolymph volume in larvae with the indicated genotypes (*n*=5/data point) **P=0.0055; ***P=0.0006; ****P<0.0001 (repeated-measures one-way ANOVA with Tukey's multiple comparisons test). Error bars in B,C indicate the mean±s.d. (D) Representative maximum-intensity projection confocal images of F-actin staining (gray), and GFP and Hipk (green) (tissues of interest are outlined with red dotted lines; *n*>4 for all discs). (E) Peristaltic movements of control, tumor-bearing and cachexic larvae within 30 s (*n*=24, 16 and 17, respectively). ns, not significant; ***P=0.0006; ****P<0.0001 (Brown–Forsythe and Welch's one-way ANOVA test with Dunnett's T3 multiple comparisons test). (F) Representative maximum-intensity projection images of abdominal segments A2-A3 of late L3 larval muscle fillets (gray, phalloidin). (G) Representative images of the medial section of ventral longitudinal muscle 3 (VL3) stained for F-actin (gray) and α-actinin (green). (H) Quantification of peak fluorescence intensities (Z-discs) of F-actin (*n*=452, 371 and 429, respectively) and α-actinin (*n*=426, 405 and 441, respectively). Box-and-whisker plot center lines show the median, box limits indicate the 25th and 75th percentiles, with whiskers extending to the minimum and maximum values in the dataset. a.u., arbitrary units. ****P<0.0001 (Brown–Forsythe and Welch's one-way ANOVA test with Dunnett's T3 multiple comparisons test). (I) Western blot of whole-protein extracts of hemolymph from third-instar larvae and probing for the presence of sarcomere protein Myosin heavy chain (Mhc). Ponceau S is used as a loading control. Images represent *n*=4 biological replicates.

Ectopic expression of Sik3-CA and Hipk in imaginal discs using the *dpp-Gal4* driver strain (*dpp>Hipk+Sik3-CA*) induces a prolonged third-instar larval phase and delayed pupariation (Yu et al., 2023). Progeny expressing Hipk and Sik3-CA continue to grow during this extended larval phase and approximately two-thirds are unable to pupariate, dying as larvae (Fig. 1A; Fig. S1A,B). To investigate this phenomenon, we chose two different developmental timepoints for the tumor model larvae. As overexpression of *Hipk* and *Sik3-CA* induces a developmental delay, we used the 50% pupariation rate to developmentally stage match with the control *dpp>GFP+RFP* larvae (Yu et al., 2023). This was to ensure that both groups are compared at equivalent developmental stages. Thus, we considered day (D) 5 control larvae to be age matched with D7-D8 *dpp>Hipk+Sik3-CA* ‘tumor’ larvae. ‘Cachexic’ larvae were defined as D16 *dpp>Hipk+Sik3-CA* larvae that were bloated (significant increase in extractable hemolymph volume) and significantly larger in mass (Fig. 1A-C). Such a tumor-induced bloating phenotype has been shown across both larval and adult *Drosophila* tumor models (Brumby, 2003; Gateff and Schneiderman, 1969; Pagliarini and Xu, 2003). Numerous *Drosophila* tumor models with this bloating phenotype have also been characterized as cancer cachexia models (Figueroa-Clarevega and Bilder, 2015; Khezri et al., 2021; Santabárbara-Ruiz and Léopold, 2021). Therefore, we hypothesized that the increase in larval mass and transparency of the larval body, indicative of loss of fat body, indicates that our *dpp>Hipk+Sik3-CA* tumor model could also induce distant organ wasting reminiscent of the human cancer cachexia-like syndrome.

Over the progression of the increased bloating from tumor bearing to cachexic, we also observed a drastic increase in tumor size and changes in epithelial tissue morphology in all epithelial discs overexpressing Hipk and Sik3-CA in the *dpp-Gal4* domain, suggesting an increase in tumor burden of the larvae over time (Fig. 1D). In these discs, either GFP or Hipk immunostaining marked the *dpp-Gal4* expression domain and labeled the tumor cells. In addition to changes in larval size, we observed significantly reduced locomotion in cachexic larvae compared to that in control and tumor-bearing larvae (Fig. 1E), with most cachexic larvae having almost no peristaltic movements within 30 s. *Drosophila* larvae move forward by peristaltic muscle contractions, and locomotion is a measure of muscle and neuronal function (Weitkunat and Schnorrer, 2014). Muscle function depends on the structure of the sarcomere, the basic repeating contractile unit of myofibers. However, we did not observe any gross abnormalities in the overall larval body wall musculature (Fig. S1F) or sarcomere protein mislocalization (Fig. 1F). In addition, the mean sarcomere length was also not significantly different from that of controls (Fig. S1D,E). This suggests that sarcomeric or muscle structure changes are not responsible for the observed locomotion defects. However, we did observe that cachexic larval muscle had reduced sarcomeric protein fluorescence intensity (Fig. 1E,F). As both F-actin and α-actinin (Actn) fluorescence intensities peak at the Z-disc, we used these as a readout of sarcomere protein levels (Fig. 1G). Both tumor and cachexic larvae had significantly reduced Z-disc fluorescence intensities compared to those of control larvae, indicating reduction of sarcomere protein in tumor-bearing larvae (Fig. 1H). In addition, cachexic larvae had significantly reduced Z-disc fluorescence intensity compared to tumor-bearing larvae, indicating a progressive loss of sarcomere proteins as the tumor progresses (Fig. 1H). Loss of sarcomere proteins is often associated with muscle wasting or degeneration; therefore, we characterized muscle wasting by probing for the major muscle component in larval hemolymph (Rodríguez-Vázquez et al., 2024). We detected Myosin heavy chain (Mhc) protein in the hemolymph of cachexic larvae but not control or tumor-bearing larvae (Fig. 1I), suggesting that cachexic larval muscle releases Mhc into circulation, likely due to muscle wasting.

As muscle mitochondria generates adenosine triphosphate (ATP) for peristaltic movement, we immunostained larval muscle to detect ATP synthase F1 subunit α (ATP5α, encoded by *bellweather* in *Drosophila*), a mitochondrial marker. Body wall muscle mitochondrial content in cachexic larvae was significantly reduced compared to that in control larvae, suggesting that reduction in mitochondria could also be contributing to reduced muscle contraction and, therefore, impaired muscle function (Fig. S1F,G).

Together, the reduction in muscle mitochondria contributing to reduced locomotion, reduction in sarcomere proteins, and secretion of muscle protein into the hemolymph suggest that *dpp>Hipk+Sik3-CA* can induce non-autonomous distant muscle wasting.

### *dpp>Hipk+Sik3-CA* induces morphological and functional changes in fat

**Fig. 2.**
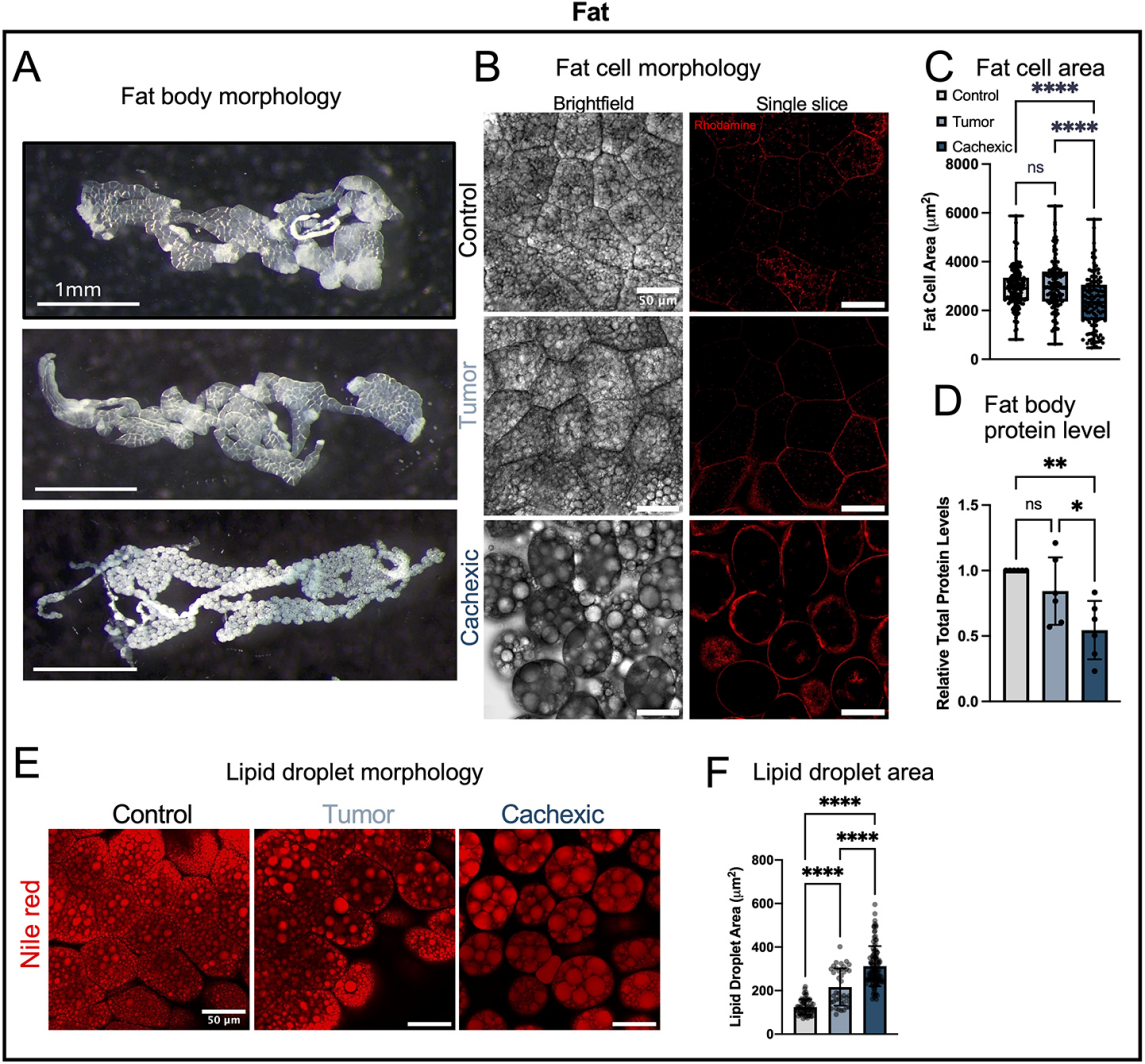
***dpp>Hipk+Sik3-CA* induces morphological and functional changes in the fat body.** (A) Dissections of fat body from indicated genotypes of third-instar larvae (*n*=10/genotype). (B) Single-slice brightfield and confocal images of F-actin staining (red) (*n*=15, 24 and 22, respectively). (C) Quantification of fat cell size (*n*=147, 118 and 116, respectively). Box-and-whisker plot center lines show the median, box limits indicate the 25th and 75th percentiles, with whiskers extending to the minimum and maximum values in the dataset. ns, not significant; ****P<0.0001 (Brown–Forsythe and Welch's one-way ANOVA test with Dunnett's T3 multiple comparisons test). (D) Quantification of total protein levels of fat body using Ponceau S staining (*n*=5 larvae/data point). Error bars indicate the mean±s.d. ns, not significant; *P=0.0424; **P=0.0095 (repeated-measures one-way ANOVA with Tukey's multiple comparisons test). (E) Representative confocal images of fat body stained with Nile Red (*n*=24, 10 and 29, respectively). (F) Quantification of the five largest lipid droplets within each immunofluorescence image (*n*=60, 40 and 105, respectively). Error bars indicate the mean±s.d. ****P<0.0001 (Brown–Forsythe and Welch's one-way ANOVA test with Dunnett's T3 multiple comparisons test).

In addition to muscle wasting, adipose tissue wasting also occurs in patients with cancer cachexia (Argilés et al., 2014). The larval fat body (considered analogous to human liver and fat tissues) is composed of a single layer of attached polygonal cells that form a large flat structure occupying the body cavity (Meschi and Delanoue, 2021). As we saw increased transparency of cachexic larvae, which is indicative of fat body loss (Fig. 1A), we wanted to characterize morphological and functional changes in the fat body that were potentially induced by the tumors. We found that cachexic larval fat bodies atrophied and dissociated into spherical cells that were significantly smaller (Fig. 2A-C). This phenotype is reminiscent of what occurs normally during *Drosophila* pupal metamorphosis, wherein Matrix metalloproteinase 1 (Mmp1) expression is induced in the third-instar larval fat body and is undetectable 6 h after pupariation, while the E-cadherin (encoded by *shotgun*)-mediated cell–cell junctions are cleaved by Mmp1, resulting in complete dissociation of the fat body into individual cells free-floating in the pupal hemolymph (Jia et al., 2014). As expected, there was a significant reduction in E-cadherin protein levels (detected by the DCAD2 antibody) in fat cell–cell junctions in cachexic larval fat cells (Fig. S2A,B). Interestingly, although there were sustained levels of Mmp1 protein at the fat cell–cell junction (Fig. S2B), transcriptional levels of *Mmp1* were significantly reduced in tumor-bearing and cachexic larval fat tissue (Fig. S2C), suggesting an alternate tissue source of Mmp1. This could potentially be mediated by significantly increased levels of Mmp1 protein from the cachexic larval tumor tissue (Fig. S2D) being secreted into the hemolymph. In addition, total protein levels of cachexic larval fat were significantly reduced compared to those of both control and tumor larval fat, indicating that there is increased catabolism (loss of tissue mass) of cachexic larval fat, resulting in tissue atrophy (Fig. 2A,D).

*Drosophila* store body fat reserves in the form of lipid droplets in the fat body cells. Due to changes in fat morphology, we hypothesized that there would also be changes in lipid droplet morphology. We found that cachexic larvae had significant lipid droplet accumulation compared to control and tumor-bearing larvae (Fig. 2E,F). As autophagy contributes to distant organ skeletal muscle wasting in patients with cancer cachexia (Aversa et al., 2016), we hypothesized that there could be increased autophagy in the larval fat body. Macroautophagy (subsequently referred to as autophagy) is a major intracellular degradation system in which autophagosomes containing cytoplasmic material are fused with lysosomes to form autolysosomes and are subsequently degraded through acidification and recycled (Mizushima and Komatsu, 2011). In *Drosophila*, late L3 larval fat undergoes upregulated programmed autophagy, and most acidic structures at this stage have been found to be autolysosomes (Rusten et al., 2004). We found that autophagic area was not significantly different between control and tumor larval fat (Fig. S2E,F). However, fluorescence intensity of the BioTracker NIR633 lysosome dye was significantly reduced in tumor larval fat compared to control larval fat (Fig. S2E,G), indicating increased autolysosomal pH, which could be due to either increased dilution of acidity resulting from more autophagic material or compromised acidification of autolysosomes. Cachexic larval fat, in contrast, showed significantly reduced autophagic area compared to both control and tumor larval fat, indicating a reduction in cachexic larval fat autophagic function as the tumor progresses (Fig. S2E,F).

### Functional changes to cachexic larval metabolism result in increased lipolysis and trehalose

**Fig. 3.**
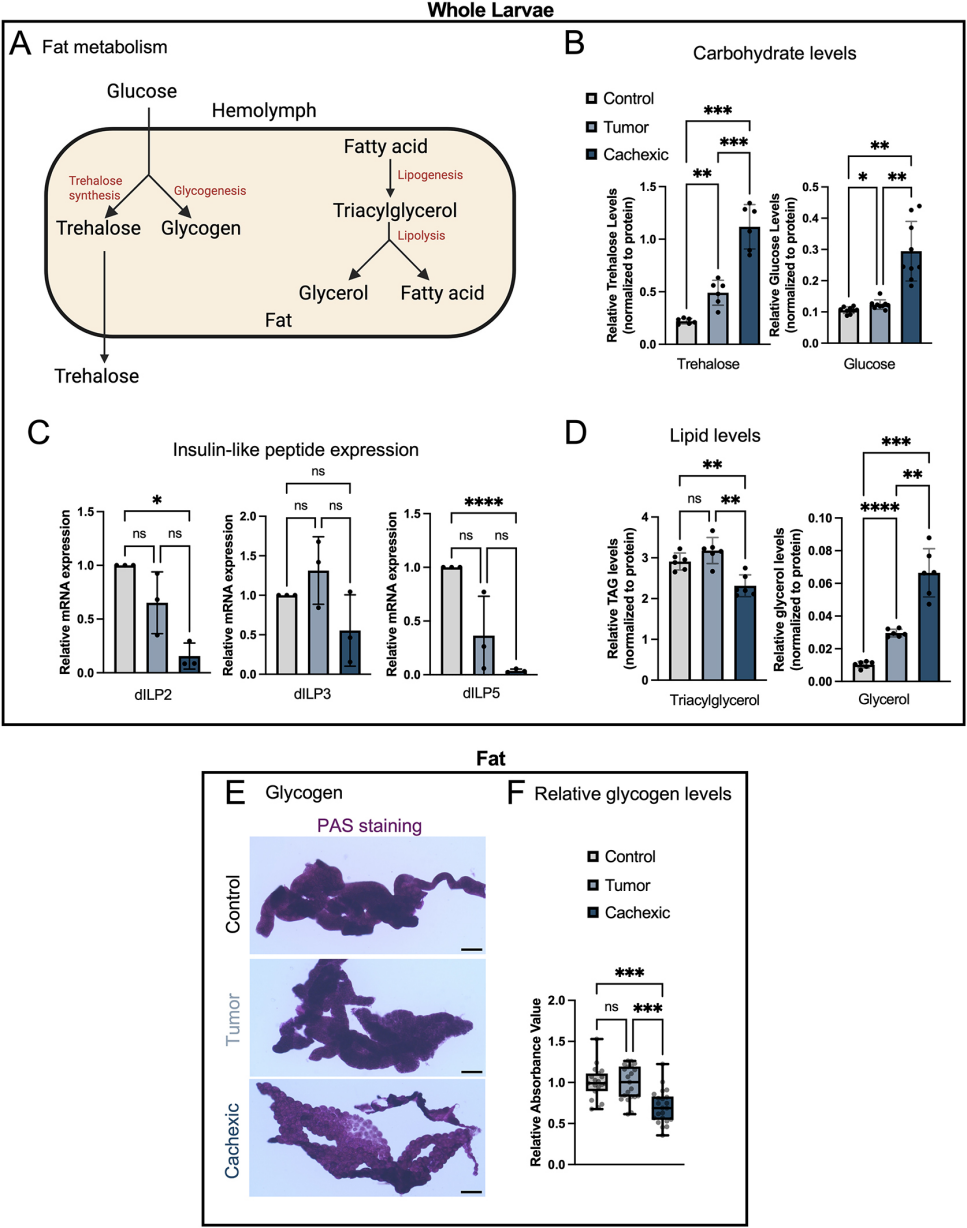
**Functional changes to cachexic larval fat results in increased lipolysis and trehalose.** (A) Schematic diagram of fat metabolism showing the hemolymph surrounding the fat body. Created in BioRender by Yu, K. (2026). https://BioRender.com/3y03cd4. This figure was sublicensed under CC-BY 4.0 terms. (B) Left: trehalose levels in whole larvae normalized to protein levels (*n*=5 larvae/data point). **P=0.0078; ***P≤0.001 (Brown–Forsythe and Welch's one-way ANOVA test with Dunnett's T3 multiple comparisons test). Right: glucose levels in whole larvae normalized to protein levels (*n*=5 larvae/data point). *P=0.0291; **P≤0.01 (Brown–Forsythe and Welch's one-way ANOVA test with Dunnett's T3 multiple comparisons test). (C) Whole-larval *dILP2, dILP3* and *dILP5* mRNA levels measured by quantitative PCR (*n*=5 larvae/data point). ns, not significant; *P=0.0125; ****P<0.0001 (repeated-measures one-way ANOVA with Tukey's multiple comparisons test). (D) Left: triacylglycerol (TAG) levels in whole larvae normalized to protein levels (*n*=5 larvae/data point). ns, not significant; **P≤0.01 (Brown–Forsythe and Welch's one-way ANOVA test with Dunnett's T3 multiple comparisons test). Right: glycerol levels in whole larvae normalized to protein levels (*n*=5 larvae/data point). **P=0.0052; ***P=0.0007; ****P<0.0001 (Brown–Forsythe and Welch's one-way ANOVA test with Dunnett's T3 multiple comparisons test). Error bars in B-D indicate the mean±s.d. (E) Representative brightfield images of fat tissue showing stored glycogen visualized by periodic acid–Schiff (PAS) staining (*n*=19, 21 and 22, respectively). Scale bars: 200 µm. (F) Relative absorbance levels of PAS staining in fat tissue. Box-and-whisker plot center lines show the median, box limits indicate the 25th and 75th percentiles, with whiskers extending to the minimum and maximum values in the dataset. ns, not significant; ***P=0.0001 (Brown–Forsythe and Welch's one-way ANOVA test with Dunnett's T3 multiple comparisons test).

The *Drosophila* fat body is the main site of metabolism, similar to the human liver (Meschi and Delanoue, 2021). Metabolism is the process of using nutrients to produce ATP, and, analogous to humans, flies use carbohydrates, lipids and proteins as energy sources (Chatterjee and Perrimon, 2021). Dietary fatty acids are taken up by the fat body and synthesized and stored as TAG, whereas hemolymph glucose is taken up and condensed into glycogen (for storage) or trehalose (a non-reducing disaccharide), and trehalose is subsequently released into the hemolymph (Chatterjee and Perrimon, 2021; Meschi and Delanoue, 2021) (Fig. 3A). Glucose and trehalose are the main circulating carbohydrates in the *Drosophila* hemolymph, with trehalose concentrations more than 100-fold those of glucose (Ugrankar et al., 2015; Wyatt, 1961). Additionally, trehalose has been found to act as a buffer for glucose homeostasis (Matsushita and Nishimura, 2020). As we observed drastic morphological changes in the fat body of cachexic larvae, we wondered whether this also resulted in metabolic dysfunction in the cachexic larvae, causing metabolite changes. Whole-larval glucose and trehalose concentrations were significantly increased in cachexic larvae (Fig. 3B). This phenotype is found in *Drosophila* diabetes models and is induced by a high sucrose diet or loss of insulin-like peptides (ILPs) (Rulifson et al., 2002; Ugrankar et al., 2015). As all the larvae were raised on the same food, yet only the tumor model developed elevated carbohydrate levels, we hypothesized that this phenotype could be mediated by loss of ILPs.

*Drosophila* ILPs have sequence similarities with human insulins, and *Drosophila* Ilp5 (Dilp5) has been shown to activate and bind to the human insulin receptor (Sajid et al., 2011). Of the *Drosophila* ILPs 1-8, Ilp2 (or dILP2), Ilp3 (or dILP3) and Ilp5 (or dILP5) are produced and secreted by insulin-producing cells in the brain and they regulate trehalose levels in *Drosophila* larvae (Nässel et al., 2015). We found that the mRNA levels of *dILP2* and *dILP5* were significantly reduced in cachexic larvae compared to control larvae (Fig. 3C). Interestingly, *dILP2*, *dILP3* and *dILP5* mutants have increased lipid and glycogen stores (Grönke et al., 2010), whereas we observed potential fat body wastingm which suggests a depletion of nutrient stores. Consistent with the fat body wasting, whole-larval TAG levels were significantly reduced and glycerol levels were significantly increased in cachexic larvae compared to both control and tumor-bearing larvae (Fig. 3D), indicating that even though *dILP2* and *dILP5* are transcriptionally repressed, lipolysis is increased through a different pathway.

Lipids exist in the form of intramyocellular lipid droplets in muscle and are a source of ATP generated from oxidative phosphorylation in muscle mitochondria. As we observed a reduction in TAG levels and mitochondrial density in cachexic larvae, we investigated muscle lipid metabolism by staining lipid droplets in the larval body wall muscle. We found an approximately 100-fold reduction in the number of lipid droplets in tumor and cachexic larval muscle (Fig. S3A,B), suggesting a reduction of lipid as a fuel source. Interestingly, even though we observed a significant reduction in intramyocellular lipid droplet number, there was a significant increase in the size of the lipid droplets (Fig. S3C); this could be due to impaired oxidative phosphorylation in tumor and cachexic larval muscle mitochondria, because we observed reduced muscle mitochondria.

In *Drosophila* larvae, glycogen is mostly stored in the brain, fat body and muscles (Yamada et al., 2018). Under starvation conditions, fat body glycogen is almost completely depleted from the fat body within 4 h to maintain circulating carbohydrate levels in the larvae, whereas no change is observed in the brain or muscle glycogen stores (Yamada et al., 2018). To visualize glycogen stored in larval fat tissue, we performed periodic acid–Schiff (PAS) staining, which results in a color reaction when bound to carbohydrates (including glycogen). We found a significant depletion of glycogen from the fat body of cachexic larvae compared to control and tumor-bearing larvae (Fig. 3E,F), suggesting that some glycogen might be broken down into glucose as the tumor progresses (glycogenolysis), potentially contributing to the high trehalose levels. Altogether, these findings reveal that tumors in epithelial discs induced by overexpression of Hipk and Sik3-CA cause distant organ wasting phenotypes in the muscle and fat body.

### Hipk+Sik-induced alterations in Lsd-2 allow elevated Brummer-mediated lipolysis

**Fig. 4.**
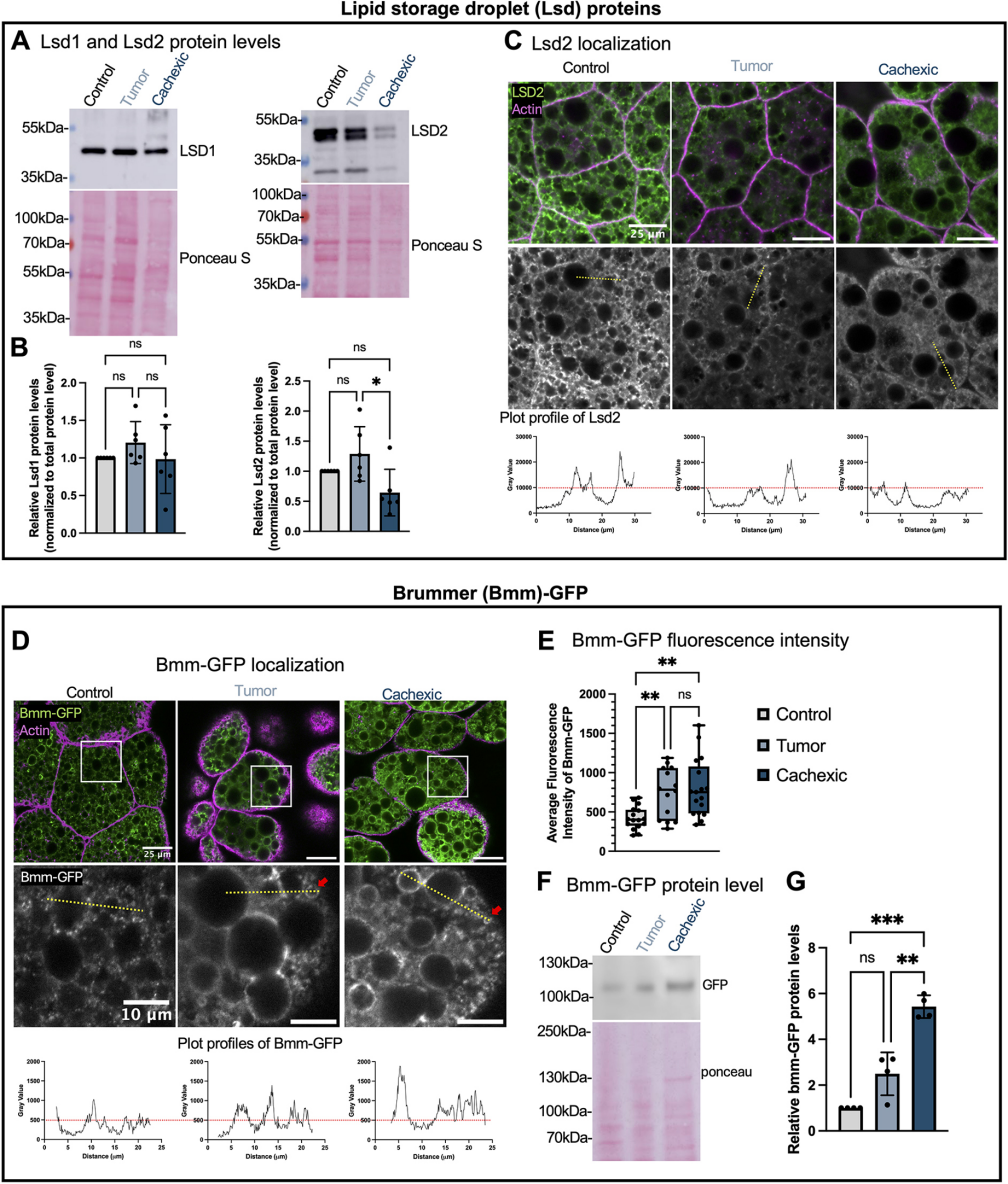
**Reduction in Lsd-2 allows Bmm–GFP more access to cachexic larval lipid droplets.** (A) Western blot of whole-protein extracts of fat tissue from third-instar larvae probed for the presence of Lsd-1 and Lsd-2 proteins. Ponceau S was used as a loading control. *n*=6 biologically independent experiments. (B) Lsd-1 and Lsd-2 protein levels were quantified and normalized to control. Ponceau S-stained membranes were used to verify total protein loading in each sample. *n*=10 larval fat bodies/data point. Error bars indicate the mean±s.d. ns, not significant; *P=0.0426 (one-way ANOVA with Tukey's multiple comparisons test). (C) Representative single-slice confocal images of F-actin staining (magenta) and Lsd-2 (green), with greyscale pictures of the Lsd-2 channel shown below. Line graphs of plot profiles (yellow dotted lines) showing the distribution of fluorescence intensity of Lsd-2 from the respective tissues are shown at the bottom (*n*=18, 14 and 16, respectively). (D) Representative single-slice confocal images of F-actin staining (magenta) and Bmm–GFP (green). Magnified sections of the Bmm–GFP channel (grey) are shown below. Higher Bmm–GFP protein accumulation around smaller lipid droplets is indicated by the red arrows. Line graphs of plot profiles (yellow dotted lines) showing the distribution of fluorescence intensity of Bmm–GFP from the respective tissues are shown at the bottom. (E) Quantification of average fluorescence intensity of fat Bmm–GFP levels (*n*=16, 14 and 17, respectively). Box-and-whisker plot center lines show the median, box limits indicate the 25th and 75th percentiles, with whiskers extending to the minimum and maximum values in the dataset. ns, not significant; **P≤0.01 (Brown–Forsythe and Welch's one-way ANOVA test with Dunnett's T3 multiple comparisons test). (F) Western blot of whole-protein extracts of fat tissue from third-instar larvae and probing for the presence of Bmm–GFP protein. Ponceau S was used as a loading control. *n*=4 biologically independent experiments. (G) Bmm–GFP protein levels were quantified and normalized to control. Ponceau S-stained membranes were used to verify total protein loading in each sample. *n*=10 larval fat bodies/data point. Error bars indicate the mean±s.d. ns, not significant; **P=0.0053; ***P=0.0008 (one-way ANOVA with Tukey's multiple comparisons test).

*Drosophila* has a dual lipolytic system: the Akh signaling pathway and the Bmm lipase lipolytic pathway (Grönke et al., 2007). Because we observed increased lipolysis in cachexic larvae, we hypothesized that one or both lipolytic systems are activated to mediate this observed depletion of TAG levels. *Drosophila* have only two perilipins, Lsd-1 (PLIN1 homolog) and Lsd-2 (PLIN2 homolog), and they have both been found to protect the lipid droplets from lipolysis mediated by Bmm lipase (Bi et al., 2012; Grönke et al., 2005). Therefore, we tested the levels of perilipins in cachexic larvae. Lsd-1 protein levels were not significantly changed in the fat body, whereas Lsd-2 protein levels were significantly reduced in cachexic larvae compared to tumor-bearing larvae (Fig. 4A,B). To determine whether reduction in Lsd-2 protein levels correlated with reduced Lsd-2 protein association with lipid droplets, we examined Lsd-2 localization in fat cells. In both control and tumor fat cells, Lsd-2 preferentially associated with smaller peripheral lipid droplets as indicated by the higher Lsd-2 fluorescence gray value peaks (Fig. 4C), consistent with previous research (Bi et al., 2012). However, in cachexic larvae, Lsd-2 appeared to have less association with peripheral lipid droplets and was more diffuse throughout the fat cell cytoplasm (Fig. 4C). As Lsd-2 protects lipid droplets from Bmm-mediated lipolysis, we examined the cellular localization of Bmm lipase with an endogenous C-terminal GFP knock-in (Zhao et al., 2022). In control larval fat, Bmm–GFP accumulated mostly around bigger lipid droplets, with low levels in the cytoplasm of fat cells (Fig. 4D). In both tumor and cachexic larval fat, Bmm–GFP accumulated more around both bigger and smaller peripheral lipid droplets, and there also appeared to be more cytoplasmic Bmm–GFP (Fig. 4D). Quantification of Bmm–GFP fluorescence intensity in fat cells was consistent with this observation and revealed that tumor and cachexic larvae had significantly more average Bmm–GFP fluorescence intensity in fat cells than control larvae (Fig. 4E). Quantification of larval fat Bmm–GFP corroborated this observation: tumor and cachexic larval fat Bmm–GFP protein levels were significantly increased compared to those in controls (Fig. 4F,G).

### Akh production is upregulated in *dpp>Hipk+Sik3-CA* tumor-bearing larvae

**Fig. 5.**
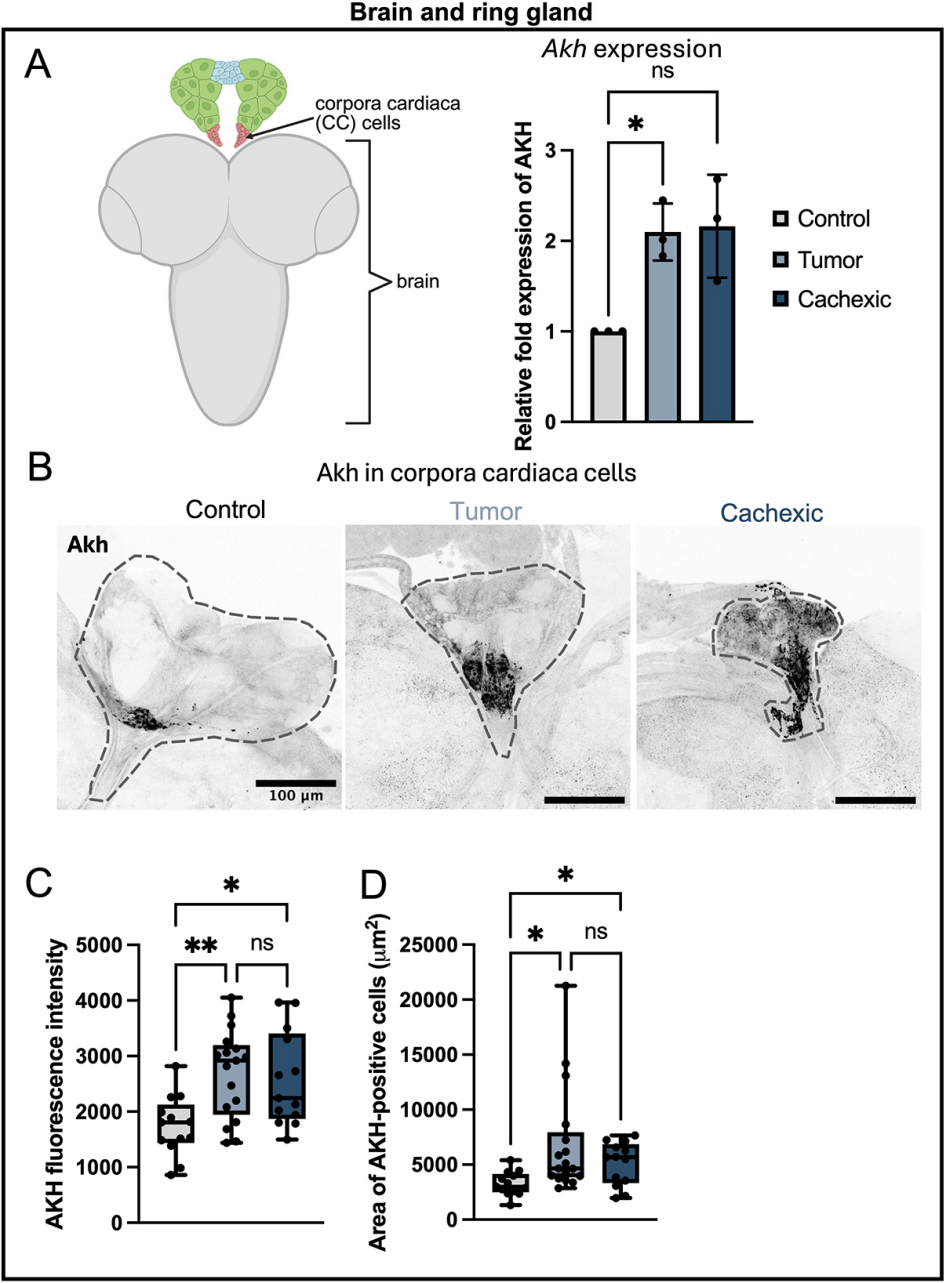
**Akh production is upregulated in *dpp>Hipk+Sik3-CA* tumor-bearing larvae.** (A) Schematic diagram of larval brain and ring gland (left) and *Akh* mRNA expression levels in brain and ring glands (right) (*n*=3 biological replicates, five flies/biological replicate). Error bars indicate the mean±s.d. ns, not significant; *P=0.0265 (unpaired two-tailed *t*-test with Welch's correction). Created in BioRender by Yu, K. (2026). https://BioRender.com/jrq2wi2. This figure was sublicensed under CC-BY 4.0 terms. (B) Maximum-intensity projection images of the ring gland (dotted region) of female L3 larvae immunostained for Akh (black, inverted lookup table). (C) Quantification of AKH fluorescence intensity (*n*=13, 13 and 17, respectively). ns, not significant; *P=0.02; **P=0.0017 (Brown–Forsythe and Welch's one-way ANOVA test with Dunnett's T3 multiple comparisons test). (D) Quantification of Akh signal area (μm^2^) (*n*=13, 13 and 17, respectively). ns, not significant; *P≤0.05 (Brown–Forsythe and Welch's one-way ANOVA test with Dunnett's T3 multiple comparisons test). Box-and-whisker plot center lines show the median, box limits indicate the 25th and 75th percentiles, with whiskers extending to the minimum and maximum values in the dataset.

*Akh* is exclusively expressed in the CC cells of the ring gland in *Drosophila* larvae (Kim and Rulifson, 2004; Lee and Park, 2004) (Fig. 5A). Ectopic expression of *Akh* induces increased lipolysis and trehalose levels in *Drosophila* larvae (Lee and Park, 2004), similar to our observations in cachexic larvae. Therefore, we examined transcription levels of *Akh* in the brain and ring gland tissue and found that *Akh* is significantly upregulated in tumor-bearing larvae (Fig. 5A). We also observed increased Akh fluorescence intensity and CC cell area in both tumor-bearing and cachexic larvae (Fig. 5B-D), indicating that there are increased Akh peptide levels in these larvae. Taken together, these results indicate that both Bmm and Akh lipolytic pathways are upregulated to induce increased trehalose and lipolysis in cachexic larvae.

Therefore, we wondered whether a reduction in the Akh lipolytic pathway would reduce the aberrant metabolism in tumor and cachexic larvae and subsequently fat wasting. Hence, we induced tumors in either a heterozygous *Akh^AP^* or *Akh receptor* (*AkhR^1^*) mutant background. Tumor and cachexic larvae heterozygous for *Akh^AP^* did not show any effects on tumor proliferation (Fig. S4A). Heterozygosity for *Akh^AP^* did significantly reduce trehalose levels in tumor larvae but did not rescue the increased trehalose and lipolysis in cachexic larvae (Fig. S4B,C). As these tumor and cachexic larvae are heterozygous for *Akh* or *AkhR*, further depletion of Akh in combination with depletion of Bmm lipase might be required to observe any significant effects. Interestingly, *AkhR^1^* heterozygous cachexic larvae were smaller and had significantly lower total protein levels than cachexic larvae (*dpp> Hipk +Sik3-CA* D16 larvae) (Fig. S4D,E), even though they had the same aberrant metabolism, specifically, increased trehalose and lipolysis (Fig. S4F,G), indicating that cachexic larval size is a separate paraneoplasia from organ wasting.

### Tumor-derived Upd ligands induce Akh production in *dpp>Hipk+Sik3-CA* tumor-bearing larvae through upregulation of the JAK/STAT signaling pathway

**Fig. 6.**
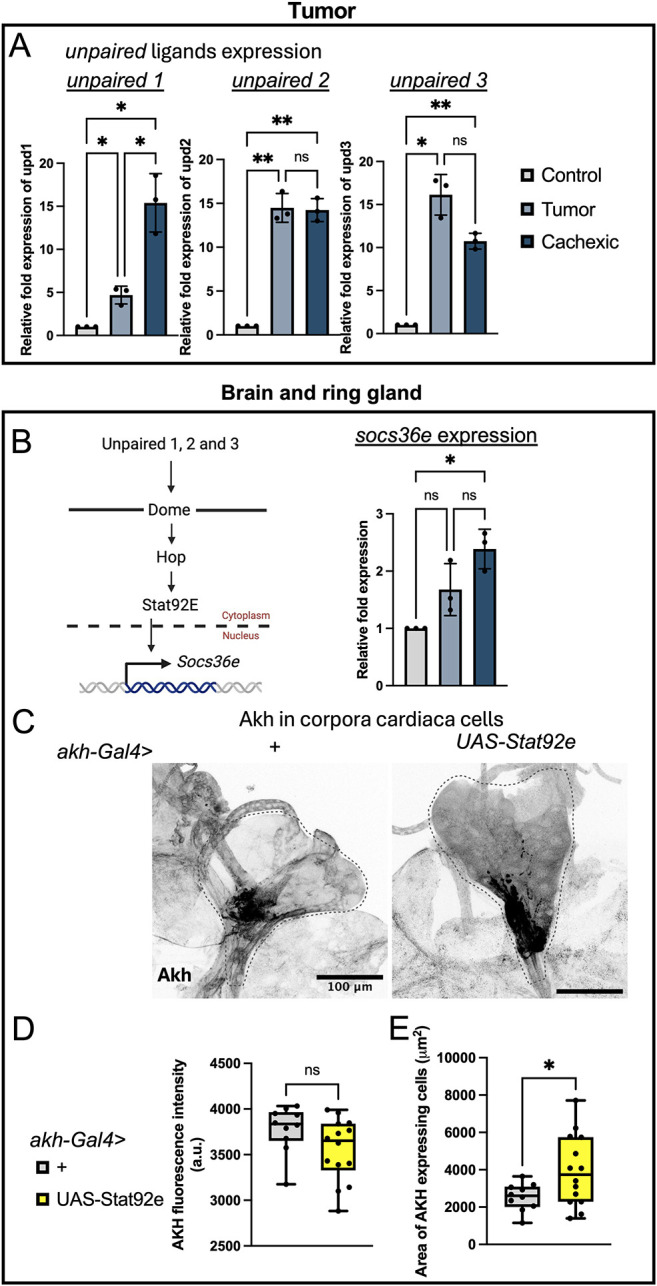
**Tumor-derived Unpaired ligands induce Akh production in *dpp>Hipk+Sik3-CA* tumor-bearing larvae through upregulation of the JAK/STAT signaling pathway.** (A) Expression levels of *unpaired 1* (*upd1*), *2* (*upd2*) and *3* (*upd1*) in epithelial discs (*n*=3 biological replicates, five flies/biological replicate). ns, not significant; *P=≤0.05; **P≤0.01 (repeated measures one-way ANOVA with Tukey's multiple comparisons test). (B) Schematic diagram of JAK/STAT signaling pathway (left) and brain and ring gland *socs36e* expression levels (right) (*n*=3 biological replicates, five flies/biological replicate). ns, not significant; *P=0.0363 (repeated-measures one-way ANOVA with Tukey's multiple comparisons test). Error bars in A,B indicate the mean±s.d. (C) Maximum-intensity projection images of the ring gland (dotted region) of female L3 larvae immunostained for Akh (black, inverted lookup table). (D) Quantification of Akh fluorescence intensity (*n*=10 and 14, respectively). ns, not significant (unpaired two-tailed *t*-test with Welch's correction). (E) Quantification of Akh signal area (µm^2^) (*n*=10 and 14, respectively). *P=0.0215 (unpaired two-tailed *t*-test with Welch's correction). Box-and-whisker plot center lines show the median, box limits indicate the 25th and 75th percentiles, with whiskers extending to the minimum and maximum values in the dataset.

Tumors use external sources of nitrogen and carbon to sustain uncontrolled cell proliferation and, therefore, metabolic reprogramming of the host organism by tumor-secreted factors to promote tissue wasting supports cancer growth (Porporato, 2016). Consequently, the tumors overexpressing Hipk and Sik-CA in our model could potentially be secreting cachexic factors to induce Akh and Bmm–GFP, resulting in increased lipolysis and the production of trehalose. A previous study in adult *Drosophila* found that the Unpaired 2 (Upd2) cytokine controls Akh secretion (Zhao and Karpac, 2017). To determine whether Upd ligands are upregulated in our tumor model, we quantified the transcription levels of genes encoding Upd ligands 1, 2 and 3 and found that they were all significantly upregulated approximately 5- to 15-fold in both tumor and cachexic larval tumors (Fig. 6A).

Upd ligands activate the JAK/STAT pathway by binding to Dome receptors, which activate the JAK Hopscotch (Hop). Activated Hop phosphorylates Stat92E, which then dimerizes and translocates to the nucleus to drive target gene expression (Harrison et al., 1998; Hou and Perrimon, 1997) (Fig. 6B). To determine whether these Upd ligands are secreted and activate the JAK/STAT pathway in CC cells, we used *socs36e* expression as a transcriptional readout of the JAK/STAT pathway and found that it was significantly induced in the brain and ring gland of cachexic larvae (Fig. 6B), indicating that the upregulation of Akh in tumor and cachexic larvae could be a result of activation of JAK/STAT signaling in CC cells. Therefore, we wondered whether knockdown of the genes encoding Upd ligands in the tumor epithelial discs could reduce secretion of Upd ligands from tumor tissue, resulting in reduced JAK/STAT activation and therefore less Akh upregulation of CC cells in tumor-bearing and cachexic larvae. Hence, we knocked down the Upd ligand genes using the *dpp-gal4* driver in the tumor background. Knockdown of *upd1* and *upd2* resulted in a slight rescue in the developmental delay phenotype (Fig. S5A). This indicates a reduction in the severity of the tumors, which is consistent with previous research in other *Drosophila* cancer models that found that Upd ligands promote overproliferation and metastasis (Ding et al., 2021; Wu et al., 2010). We used this pupariation curve to developmentally stage match the larvae for more accurate comparison. We found that *upd3* knockdown in tumors, but not *upd1* or *upd2* knockdown, significantly reduced the Akh signal area compared to that in the tumor condition alone (Fig. S5B,C), indicating that Upd3 is potentially secreted from tumors and activates JAK/STAT in CC cells to upregulate Akh protein levels. Finally, overexpression of the JAK/STAT transcription factor Stat92E using a CC specific *akh-Gal4* driver resulted in increased Akh signal area (Fig. 6C-E), consistent with results found in adult *Drosophila* linking JAK/STAT signaling to Akh production (Zhao and Karpac, 2017).Taken together, these results suggest that the tumors secrete Upd ligands into the hemolymph and activate JAK/STAT signaling pathway in CC cells, inducing increased Akh peptide levels and promoting lipolysis.

## DISCUSSION

**Fig. 7.**
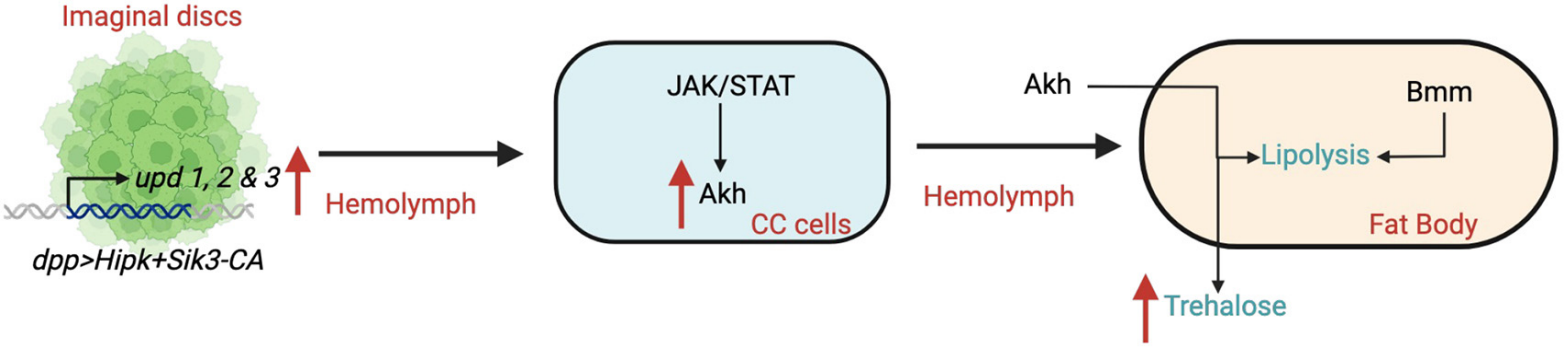
**Model of changes in distant organ functions and metabolite changes induced by overexpression of *Hipk* and *Sik3-CA* in larval epithelial discs.** Imaginal discs overexpressing *Hipk* and *Sik3-CA* upregulate the secretion of Unpaired (Upd) ligands 1, 2 and 3. These ligands are potentially secreted into the hemolymph to activate JAK/STAT signaling in distant organ corpora cardiaca (CC) cells, resulting in increased Akh protein levels. Akh is then potentially secreted and, together with Bmm lipase, increases lipolysis in the distant organ fat body. Akh also results in increased trehalose synthesis in the fat body. Created in BioRender by Yu, K. (2026). https://BioRender.com/p820u09. This figure was sublicensed under CC-BY 4.0 terms.

In this study, we show that overexpression of Hipk and Sik3-CA in epithelial tissues of *Drosophila* larvae produce a *Drosophila* cancer cachexia model. We also demonstrate tumor–peripheral organ communication that results in changes in host metabolism. In particular, our results identify an Upd–Akh signaling axis that contributes to increased trehalose levels and lipolysis in cachexic larvae (Fig. 7). As the *Drosophila* JAK/STAT and glucagon-like Akh signaling pathways are highly conserved in humans, we believe that this *Drosophila* cancer cachexia model could provide more insight into the complex metabolic changes in human cancer cachexia.

We started this line of investigation due to the developmental delay phenotype that we saw in our cancer cachexia model, which is also commonly seen in other *Drosophila* cancer and cancer cachexia models (Liu et al., 2022; Menut et al., 2007). It has been established that this developmental delay is caused by an upregulation of insulin-like peptide 8 (Ilp8 or dILP8) and Upd ligands in response to injury or tumor, which inhibits ecdysone synthesis, hence delaying metamorphosis (Cao et al., 2022; Colombani et al., 2012; Garelli et al., 2012). From various studies, developmental delay by itself is not sufficient to induce distant organ wasting (Hodgson et al., 2021), and reduction or silencing *dILP8* in *Drosophila* cancer cachexia models rescues developmental delay without rescuing cancer cachexia (Santabárbara-Ruiz and Léopold, 2021; Yeom et al., 2021). Therefore, developmental delay and cancer cachexia are separate paraneoplasias.

We found muscle dysfunction and wasting in our cancer cachexia model, consistent with the *scrib Ras^V12^* and *yki^act^ Drosophila* cancer cachexia models (Figueroa-Clarevega and Bilder, 2015; Kwon et al., 2015). This is commonly found in patients with cancer cachexia as well, and it is usually caused by multiple interconnected signaling pathways that converge to induce muscle wasting through activation of catabolic pathways (Rausch et al., 2021; Setiawan et al., 2023). Of relevance, muscle mitochondrial dysfunction is commonly associated with cancer cachexia (Setiawan et al., 2023). Interestingly, excessive fatty acid oxidation in mouse skeletal muscle was found to induce muscle wasting under a lipid-induced insulin resistance disease condition (Koves et al., 2008). Additionally, in the *Ras^V12^ dlg^RNAi^ Drosophila* cancer cachexia model, muscle wasting was mediated by increased lipid utilization due to mitochondrial fusion (Dark et al., 2024). This could explain why we found reduced numbers of lipid droplets in the tumor and cachexic larval muscle – due to increased breakdown of lipids into fatty acids and subsequent fatty acid oxidation. It would be interesting to test whether in our model the larval muscle is also insulin resistant. In contrast to our findings, studies in human, mouse and *Drosophila* muscle have found increased number and size of lipid droplets in cancer cachexia (Dark et al., 2024; Hodgson et al., 2021; Stephens et al., 2011). This increase in lipid droplets is hypothesized to be due to reduced fat oxidation in muscle mitochondria. Either way, both these phenotypes point to mitochondrial dysfunction in cachexic muscle. In addition, IL6, the mammalian homolog of Upd3, is a known myokine that induces muscle wasting (Agca and Kir, 2024; Muñoz-Cánoves et al., 2013). This is also consistent with the *yki^act^ Drosophila* cancer cachexia model in which the authors showed that gut tumors secrete Upd3 to induce muscle wasting (Ding et al., 2021).

We found the cachexic larval fat body to be the central regulator of metabolite changes in this *Drosophila* cancer cachexia model. Morphologically, we found rounding of fat cells, potentially due to Mmp1 secreted from tumor tissues resulting in reduction of E-cadherin in fat cell–cell junctions. Additionally, this fat body cell rounding has also been observed in another *Drosophila* cancer cachexia model, in which tumor-secreted Mmp1 disrupts the basement membrane and extracellular matrix proteins at the adipocyte cell–cell adhesion junctions, resulting in rounding of fat cells (Lodge et al., 2021). However, in a recently published article, suppression of E-cadherin in fat cells did not result in fat cell rounding (Almasoud et al., 2026), indicating that Collagen IV may be the sole mediator of cell–cell adhesion in *Drosophila* larval fat cells. This suggests that, in this cancer cachexia model, fat cell rounding could be due to Mmp1 degrading Collagen IV instead of E-cadherin. We also found increased lipid droplet size associated with tumor and cachexic larvae, consistent with findings from the *scrib Ras^V12^* and *Ras^V12^ dlg^RNAi^ Drosophila* cancer cachexia models (Figueroa-Clarevega and Bilder, 2015; Lodge et al., 2021). Of note, reduction in Lsd-2 (PLIN2) and increased Bmm (ATGL) results in smaller lipid droplets due to increased lipolysis (Beller et al., 2010; Grönke et al., 2005). Lipid droplet size is determined by the lipid content, composition of the phospholipid monolayer and lipid droplet-associated proteins or perilipins (Zhao et al., 2022). This suggests that the larger lipid droplets we observed in our cachexia model are the result of changes to lipid content or phospholipid composition. We also found fat tissue atrophy and reduced fat protein levels, indicative of tissue catabolism in cachexic larval fat. This is consistent with phenotypes in patients with cancer cachexia, in whom weight loss mainly results from catabolism of adipose and skeletal muscle (Pryce et al., 2023). However, autophagy appears to be compromised in cachexic larval fat. This could be due to reduced ecdysone signaling, as a previous study has found that ecdysone signaling induces programmed autophagy in L3 larval fat (Rusten et al., 2004) and, in our *Drosophila* cancer cachexia model, we saw a developmental delay caused by reduced ecdysone signaling. Further experiments would be needed to determine whether reduced ecdysone signaling is the main cause of the reduced autophagy we see in cachexic larval fat. In addition, another major protein catabolism pathway, the ubiquitin ligase proteasome pathway, could also be responsible for the fat tissue catabolism in cachexic larvae.

Metabolically, functional changes such as increased carbohydrates and lipolysis are consistent with other *Drosophila* and mice cancer cachexia models, and phenotypes in human patients (Agustsson et al., 2007; Berriel Diaz et al., 2024; Dev et al., 2018; Rydén et al., 2008). In patients with cancer cachexia, the increase in glucose is proposed to be a result of lactate secreted from tumor cells leading to increased hepatic glucose production (Masi and Patel, 2021). Additionally, adipose tissue loss in human patients is a result of increased lipolysis and lipid utilization, and potentially impaired lipogenesis (Fang et al., 2023; Rydén et al., 2008). Another metabolic change we found was a significant reduction in glycogen storage in cachexic larval fat tissue. Research on this energy storage molecule is non-existent in patients with cancer cachexia and minimally investigated in one *Drosophila* and one mouse cancer cachexia model. Consistent with our findings in this paper, the *yki^act^ Drosophila* cancer cachexia model found significantly reduced total glycogen levels (Kwon et al., 2015). Furthermore, a mouse cancer cachexia model also showed a progressive reduction in glycogen in the liver of cachexic mice (Hirai et al., 1997). It would be interesting to investigate glycogen levels in patients with cancer cachexia as glycogen is the main form of glucose storage in animals and cancer cachexia is a metabolic disease.

Of note, insulin resistance is a common comorbidity in cancer cachexia models and patients (Dev et al., 2018; Figueroa-Clarevega and Bilder, 2015; Khezri et al., 2021; Kwon et al., 2015). However, in our model, we found that hyperglycemia is caused by a reduction in ILPs or a suppression of the insulin pathway. It should be noted that in a study with over 600 patients with cancer, over a third showed insulin resistance (Glicksman and Rawson, 1956), indicating that around two thirds of patients did not have insulin resistance, pointing to the heterogeneity of cancer effects. This highlights the necessity of characterizing different models of cancer cachexia as human cancer cachexia is a complicated and heterogenous disease.

We found that elevated Bmm–GFP and Akh mediated increased lipolysis in cachexic larvae (Fig. 7). This is consistent with a mouse model of cancer cachexia in which the authors found that *Atgl*-deficient mice with tumors rescued the increased lipolysis and adipose tissue and muscle wasting (Das et al., 2011). To further address mechanisms underlying the observed phenotypes, future experiments could incorporate an additional expression system along with UAS-Gal4 to induce a fat-specific knockdown of *bmm* in cachexic larvae to investigate any reduction in lipolysis and rescue of distant organ-wasting phenotypes. As we also found increased Akh in tumor and cachexic larvae, there are also numerous mouse and human cancer cachexia studies showing increased glucagon plasma levels (Bartlett et al., 1995; Ding et al., 2025). Our model shows that Upd ligands upregulate the JAK/STAT signaling pathway in CC cells and subsequently upregulate Akh to induce lipolysis (Fig. 7). Recently, another study also showed that Akh is essential for host distant organ wasting in the *yki^3SA^* gut tumor model, consistent with our findings (Ding et al., 2025). However, the authors found that Pvf1 to be the cachexic factor mediating Akh upregulation (Ding et al., 2025). Given that tumors have been shown to secrete numerous cachexic factors to promote distant organ wasting (Liu et al., 2022; Setiawan et al., 2023), multiple factors could be contributing to the same phenotype. Furthermore, Upd3/IL6 are often associated with cancer cachexia in *Drosophila*, mice and humans (Bonetto et al., 2012; Ding et al., 2021). Although these studies have mostly focused on muscle wasting induced by Upd3/IL6 inflammation, our study shows a previously unreported connection between Upd ligands activating lipolysis through Akh. Outside of the context of cancer cachexia, this result is consistent with human and mouse research, which found that IL6 stimulates glucagon secretion (Chow et al., 2014). It would be interesting to know whether in the context of cancer cachexia, IL6 can also stimulate glucagon secretion in human patients.

While trying to reduce Akh signaling and, therefore, lipolysis by inducing the Hipk+Sik tumor in heterozygous *AkhR* mutants, we observed that the cachexic larvae in the heterozygous *AkhR* mutant background were smaller in size and, therefore, had significantly lower protein levels compared to control cachexic larvae. However, the reduction in size had no effect on the increased lipolysis and trehalose levels, indicating that increased larval size in this context is a separable non-autonomous effect from organ wasting. Regulation of larval body or organ size is dependent on environmental factors, such as nutrition and temperature, and inter-organ coordination occurs mostly through hormones and secreted proteins (Mirth and Shingleton, 2012). Adult *AkhR* mutants do not have any significant changes in size (Bharucha et al., 2008); therefore, the reduction in *AkhR* in cachexic larvae is not likely to contribute to smaller larvae. Ablation of insulin-producing cells that secrete the dILP2, dILP3 and dILP5 reduced larvae size (Rulifson et al., 2002), which is inconsistent with our cancer cachexia model as we saw reduced *dILP2* and *dILP5* expression levels, suggesting that the reduction in cachexic larval size is due to other factors.

Our study shows that tumors secrete Upd ligands to upregulate Akh in CC cells, resulting in increased trehalose and lipolysis in the cachexic larvae. Tumors use external sources of nitrogen and carbon to sustain uncontrolled cell proliferation and, therefore, metabolic reprogramming of the host organism by tumor-secreted factors to promote tissue wasting supports cancer growth (Porporato, 2016). Hence, we speculate that this organismal change in metabolites (increase in carbohydrates and fatty acids) feeds into tumor growth. Thus, we are currently investigating the energy metabolism of tumors, such as rates of glycolysis and oxidative phosphorylation. Of note, a previous study suggests that Sik3 acts cell-autonomously downstream of both the insulin and Akh pathways to regulate *bmm* expression in the fat body (Choi et al., 2015). Please note that, in our study, Sik3-CA is overexpressed in the epithelial discs and not in the fat body; therefore, Sik3-CA is regulating insulin and the Akh pathway potentially with Hipk non-autonomously. It would be of interest to test whether *Sik3-CA* overexpression in the tumors increases *bmm* expression and, if so, whether the tumor utilizes the fatty acid released from lipolysis for increased proliferation. In addition, the increased glutamine consumption by cancer cells is correlated with Myc activation in human cancer cell lines and mouse cancer models (Zheng, 2012). As we also found increased dMyc protein levels in the Hipk+Sik tumors (Yu et al., 2023), we could test whether there is also increased glutamine consumption contributing to the increased cell proliferation mediated by increased levels of dMyc.

## MATERIALS AND METHODS

### *Drosophila* culture

*Drosophila melanogaster* flies were raised on standard cornmeal-molasses food. Stocks were kept at 18°C or room temperature. Crosses were carried out at 29°C as indicated. The following fly strains were used: *dpp-Gal4/TM6B* (Staehling-Hampton et al., 1994), *akh-gal4* [Bloomington *Drosophila* Stock Center (Bloomington, IN, USA) (BDSC), #25684], *UAS-GFP* (BDSC, #5431), *UAS-RFP* (BDSC, #7119), *UAS-Hipk^3M^* (Blaquiere et al., 2018), *UAS-Sik3-CA* (Wehr et al., 2013), Bmm-GFP (Zhao et al., 2022), *w^1118^* (BDSC, #5905), *UAS-Stat92E-GFP* (Sotillos et al., 2013), *Akh^AP^* (Gáliková et al., 2015), *AkhR1* (Grönke et al., 2007), *UAS-unpaired1-RNAi* (BDSC, #33680), *UAS-unpaired2-RNAi* (BDSC, #33988) and *UAS-unpaired3-RNAi* (BDSC, #32859).

### Larval mass and hemolymph volume

To measure larval mass and hemolymph volume, we adapted the protocol from Santabárbara-Ruiz and Léopold (2021). Third-instar larvae were first rinsed in 1× PBS and dried on a Kimwipe. They were then weighed in groups of five to determine mass. Next, the groups of five larvae were transferred to parafilm and abdominal openings were made with forceps, allowing the hemolymph to pool out surrounding the larvae. Hemolymph was then extracted with a pipette tip.

### Pupal counting

Flies were allowed to lay eggs for 24 h. We then counted number of pupae on the walls of the food vials of our genotypes of interest that pupated over 14 days.

### Immunohistochemistry

Imaginal discs from late L3 larvae were dissected in PBS and fixed in 4% paraformaldehyde (PFA) for 15 min at room temperature. After fixation, samples were washed in PBS with 0.1% Triton X-100 (PBST). After blocking with 5% bovine serum albumin (BSA) in PBST for 1 h at room temperature, samples were incubated with primary antibodies overnight at 4°C.

The protocol for larval body wall muscle dissection was adapted from Dark et al. (2022). Third-instar larvae were washed in PBS and subsequently heat killed in 70-75°C PBS for 5 s. Larval muscle fillets were dissected and fixed in ice-cold 4% PFA for 20 min at room temperature. To stain for lipid droplets in muscle, larval body wall muscle was incubated overnight at 4°C in Bodipy 493/503 (Invitrogen, D3922).

The protocol for larval fat dissections was adapted from Dark et al. (2022). Third-instar larvae were dissected in PBS and fixed in 4% PFA for 30 min at room temperature. To stain for lipid droplets, fixed fat tissue was incubated for 30 min at room temperature in Nile Red dye (1:500, Invitrogen, N1142). After mounting, fat samples were imaged immediately or within the same day.

The primary antibodies used include rabbit anti-Hipk (1:200; Blaquiere et al., 2018), mouse anti-ATP5α (1:500; Abcam, ab14748), rat anti-E-cadherin [1:100; Developmental Studies Hybridoma Bank (DSHB), DCAD2, AB_528120)], mouse anti-Mmp1 (1:100; DSHB, 3A6B4, 3B8D12 and 5H7B11 used together), rabbit anti-Lsd-2 (1:500; gift from Dr Ronald Kühnlein, Institute of Molecular Biosciences, University of Graz, Graz, Austria), rabbit anti-GFP (1:500; Thermo Fisher Scientific, A11122), rabbit anti-Akh (1:500; gift from Dr Wei Song, Wuhan University, Wuhan, Hubei, China) and mouse anti-α-actinin (1:50; DSHB, 2G3-3D7).

After washing with PBST (PBS for Nile Red staining and PBS in 0.05% saponin for Bodipy staining of muscle), samples were incubated with FITC- and/or Alexa Fluor 647-conjugated secondary antibodies (1:500; Jackson ImmunoResearch Laboratories, 711-095-152, 711-605-152, 712-605-153 and 715-095-151). DAPI (Invitrogen, D1306) was used at 1:500 and Phalloidin-Rhodamine (Invitrogen, R415) was used at 1:1000. Samples were mounted in VECTASHIELD Antifade Mounting Medium (Vector Laboratories, H-1000-10) after washing. Images were acquired on a Zeiss LSM880 Airyscan confocal microscope and processed using ImageJ (Schindelin et al., 2012).

### Locomotion assay

We adapted the protocol from Fushiki et al. (2016). Third-instar larvae were rinsed in 1× PBS, dried on a Kimwipe, then transferred to an agar plate and acclimatized for 2 min. The larvae were then recorded using an iPhone 14 Pro. The peristaltic movement was manually counted in the videos over 30 s.

### Sarcomere size quantification

We adapted the protocol from Zapater et al. (2023). For F-actin sarcomere length quantifications, three lines were drawn across the ventral longitudinal muscle 3 (VL3) in the anterior, medial and posterior regions spanning several Z-discs. Subsequently, a plot profile was generated from each line and the *x*-coordinates of the peaks (Z-discs) were acquired with the ImageJ/Fiji plugin BAR (https://doi.org/10.5281/zenodo.28838). The distance between six peaks was obtained by calculating the distance between the *x*-coordinates. The average value from the three lines was used as one sarcomere size data point. One to four sarcomere data points were obtained from each larva. For α-actinin sarcomere length quantifications, one line was drawn across the entire VL3 (medial portion). One to two sarcomere data points were obtained from each larva.

### Z-disc fluorescence intensity measurement

For both F-actin and α-actinin Z-disc fluorescence intensity measurements, one line was drawn across the entire VL3 (medial portion). Subsequently, a plot profile was generated and the gray value of the peaks (Z-discs) were acquired with the ImageJ/Fiji plugin BAR. 14-20 peak values were obtained from each muscle.

### Western blotting

Hemolymph extracted from five late-third-instar female larvae was diluted 10× in 5× Laemmli buffer. Cells or tissues were lysed with 1× cell lysis buffer (Cell Signaling Technology, 9803), supplemented with 1× protease inhibitors (Roche) and 1 mM phenylmethylsulfonyl fluoride. All samples were heated at 95°C for 5 min.

Lysates were stored at −20°C. Protein lysates were resolved in 4-15% gels by SDS-PAGE (at 90 V for 120 min) and then transferred to nitrocellulose membranes (at 20 V for 65 min). For Ponceau S staining, nitrocellulose membranes were incubated in Ponceau S solution [40% methanol (v/v), 15% acetic acid (v/v), 0.25% Ponceau S] for 5 min at room temperature. The membranes were destained using TBS containing 0.1% Tween 20 detergent (TBST). Membranes were blocked with 5% BSA in TBST before primary and secondary antibody incubation. Images were acquired using a FujiFilm LAS-4000 Chemiluminescent Scanner.

The primary antibodies used were: mouse anti-Mhc (1:500; DSHB, BB7/86.1), mouse anti-Mmp1 (1:500; DSHB, 3A6B4, 3B8D12 and 5H7B11 used together), rabbit anti-Lsd-1 (1:3000) and rabbit anti-LSD2 (1:5000) (gifts from Dr Ronald Kühnlein), and rabbit anti-GFP (1:5000; Thermo Fisher Scientific, A11122).

### Live BioTracker NIR633 lysosome dye assay

Larval fat bodies were dissected in ice-cold PBS and incubated with BioTracker NIR633 lysosome dye (1:1000; Sigma-Aldrich, SCT138) for 15 min at room temperature to label acidic organelles including autolysosomes. Tissues were then washed in ice-cold PBS once for 5 min, mounted and imaged immediately.

### Quantification of BioTracker NIR633 lysosome dye

The area of each fat cell was outlined in brightfield images. Organelles positive for BioTracker NIR633 lysosome dye were outlined using the Analyze Particles tool (particles >0.1 μm^2^) in ImageJ after thresholding maximum-intensity projection images to obtain area and average fluorescence intensity.

### Measurement of carbohydrate and lipid levels

Five female larvae were homogenized in 500 μl PBST. The solution was incubated at 75°C for 5 min, then spun down at 16,000 ***g*** for 10 min at 4°C. The supernatant was collected and used to perform glucose (Autokit Glucose, FujiFilm, 997-03001), trehalase (MilliporeSigma, T8778-1UN), TAG (Triglyceride Colorimetric Assay Kit, Cayman Chemical, 10010303-96), glycerol (Glycerol Colorimetric Assay Kit, Cayman Chemical, 10010755-96) and Bradford (Pierce BCA Protein Assay Kit, Thermo Fisher Scientific, 23225) assays according to the manufacturers’ instructions.

### Quantitative real-time PCR

Whole larvae from the third-instar larval stage or specific tissues dissected from third-instar larvae were briefly washed in PBS and temporarily stored in RNAlater Stabilization solution (Invitrogen, AM7020). Total RNA was extracted using RNeasy Mini Kits (Qiagen, 74101). The first strand of cDNA was synthesized using the OneScript Plus cDNA Synthesis Kit (Applied Biological Materials, G236). Quantitative real-time PCR was performed using the StepOne Real-time PCR System (Applied Biosystems). The primers used were: *Dilp2* F, 5ʹ-ACGAGGTGCTGAGTATGGTGTGC-3ʹ; *Dilp2* R, 5ʹ-CACTTCGCAGCGGTTCCGATATCG-3ʹ; *Dilp3* F, 5ʹ-GTCCAGGCCACCATGAAGTTGTGC-3ʹ; *Dilp3* R, 5ʹ-CTTTCCAGCAGGGAACGGTCTTCG-3ʹ; *Dilp5* F, 5ʹ-TGTTCGCCAAACGAGGCACCTTGG-3ʹ; *Dilp5* R, 5ʹ-CACGATTTGCGGCAACAGGAGTCG-3ʹ; *MMP1* F, 5ʹ-AGGACTCCAAGGTAGACACAC-3ʹ; *MMP1* R, 5ʹ-TTGCCGTTCTTGTAGGTGAACGC-3ʹ; *Akh F*, 5ʹ-TCCCAAGAGCGAAGTCCTCA-3ʹ; *Akh R*, 5ʹ-CCAGAAAGAGCTGTGCCTGA-3ʹ; *Unpaired 1 F*, 5ʹ-TGGATCGACTATCGCAACTTCG-3ʹ; *Unpaired 1 R*, 5ʹ-GTCCTGGCTACTGTTTAGGCT-3ʹ; *Unpaired 2 F*, 5ʹ-GAGGGCAGCTACGACAGTG-3ʹ; *Unpaired 2 R*, 5ʹ-GGAGAAGAGTCGCAGGTTGT-3ʹ; *Unpaired 3 F*, 5ʹ-CTGGTCACTGATCTTACTCGCC-3ʹ; *Unpaired 3 R*, 5ʹ-GGATTGGTGGGATTGATGGGA-3ʹ; *Socs36e F*, 5ʹ-ATGGGTCATCACCTTAGCAAGT-3ʹ; and *Socs36e R*, 5ʹ-TCCAGGCTGATCGTCTCTACT-3ʹ.

### Histochemistry

Larval fat bodies were dissected in PBS and fixed in 4% PFA for 20 min at room temperature. Tissues were then washed in PBS and incubated with periodic acid for 5 min. To stain for glycogen, fixed fat tissues were incubated for 30 min at room temperature in Schiff's reagent. After mounting, fat samples were imaged immediately or within the same day.

### Semi-quantitative measurement of glycogen levels

The fat tissues stained with periodic acid-Schiff (PAS) were imaged with a Zeiss Axioplan 2 microscope under bright field. Images for each biological replicate were acquired with identical illumination settings. To quantify the PAS signal intensity from the fat tissue images, the Colour Deconvolution 2 ImageJ plugin was used (using the built-in H PAS vector) to obtain the absorbance value of the PAS staining (Landini et al., 2021; Ruifrok and Johnston, 2001). Relative absorbance value was quantified by normalizing all absorbance values by the average of control sample values.

### Statistical analyses

All statistical analyses were performed using GraphPad Prism 10.3.1 (GraphPad Software, San Diego, USA). Box-and-whisker plot center lines show the median, box limits indicate the 25th and 75th percentiles, with whiskers extending to the minimum and maximum values in the dataset. Analyzed data with *P*<0.05 were considered statistically significant.

## Supplementary Material

10.1242/dmm.052659_sup1 Supplementary information
