# Supplementary material for: A Drosophila tumor model identifies a conserved Upd–JAK/STAT–Akh signaling axis associated with metabolic changes in cancer cachexia
Source: Dis Model Mech. 2026 Jun 16;19(7):dmm052659. doi: 10.1242/dmm.052659 (PMC13312922; doi:10.1242/dmm.052659)
Supplement: Supplementary information [file dmm-19-052659-s1.pdf]

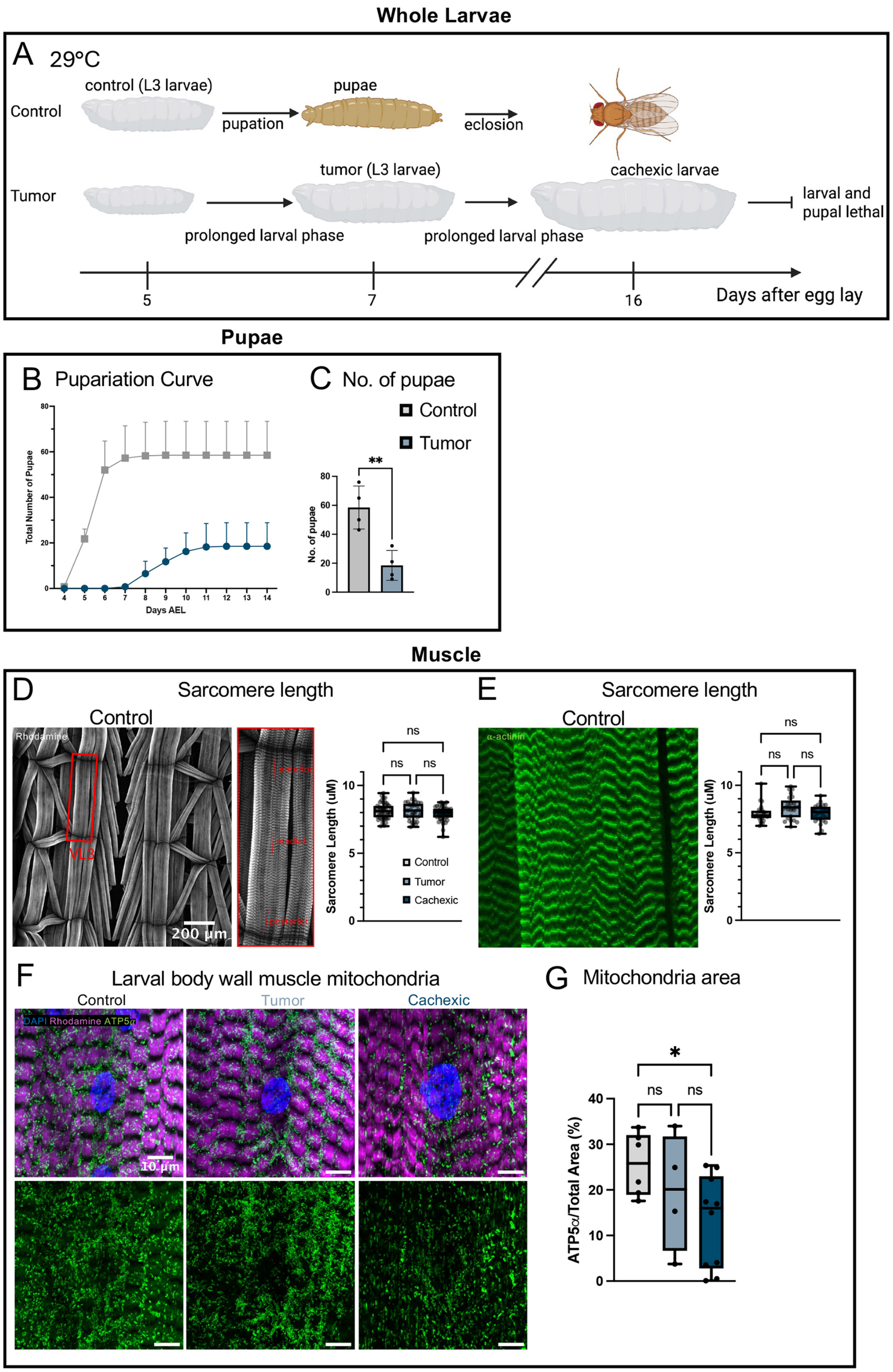

Disease Models & Mechanisms • Supplementary information

Fig. S1. Overexpression of Hipk and Sik-3CA significantly reduces the number of pupae.

(A) Schematic diagram depicting development of control and tumor-bearing larvae at 29°C. Quantification of number of pupae of the indicated genotypes. (n=4 biological replicates) ∗∗p = 0.0058 (Welch's t-test). Created in BioRender by Yu, K. (2026). https://BioRender.com/6dbgw6d. This figure was sublicensed under CC-BY 4.0 terms.

(B) Cumulative number of pupae (mean ± standard deviation) days after egg lay (AEL) of the indicated genotypes. (n=4 biological replicates)

(C) Quantification of total cumulative number of pupae of the indicated genotypes. ∗∗p = 0.0058 (Welch's t-test).

(D) VL3 muscle in A2 segment of control larva (red box) (image is identical to control image in Fig. 1F). Red lines across the VL3 muscle (right) show the anterior, medial and posterior regions used to quantify sarcomere length of each muscle. Quantification of sarcomere length (n=46, 34 and 42 respectively) ns>0.9999, =0.2746 and 0.4004 respectively (Brown-Forsythe and Welch ANOVA test)

(E) Medial region of VL3 muscle (left) (image is identical to control image in Fig. 1G). Quantification of medial region of VL3 muscle sarcomere length (n=26, 25 and 25 respectively) (right) ns=0.0842, 0.9999 and 0.0843 respectively (Brown-Forsythe and Welch ANOVA test)

(F) Longitudinal maximum projection confocal images of ventral longitudinal VL4 from A2-A4 hemisegments. Anti-ATP5α (green), F-actin (red), and DAPI (blue). (n=7, 4 and 9 respectively)

(G) Quantification of area of ATP5α as a percentage of total area of muscle. ns=0.775 and 0.7591 respectively, and ∗p = 0.00293 (Brown-Forsythe and Welch ANOVA test)

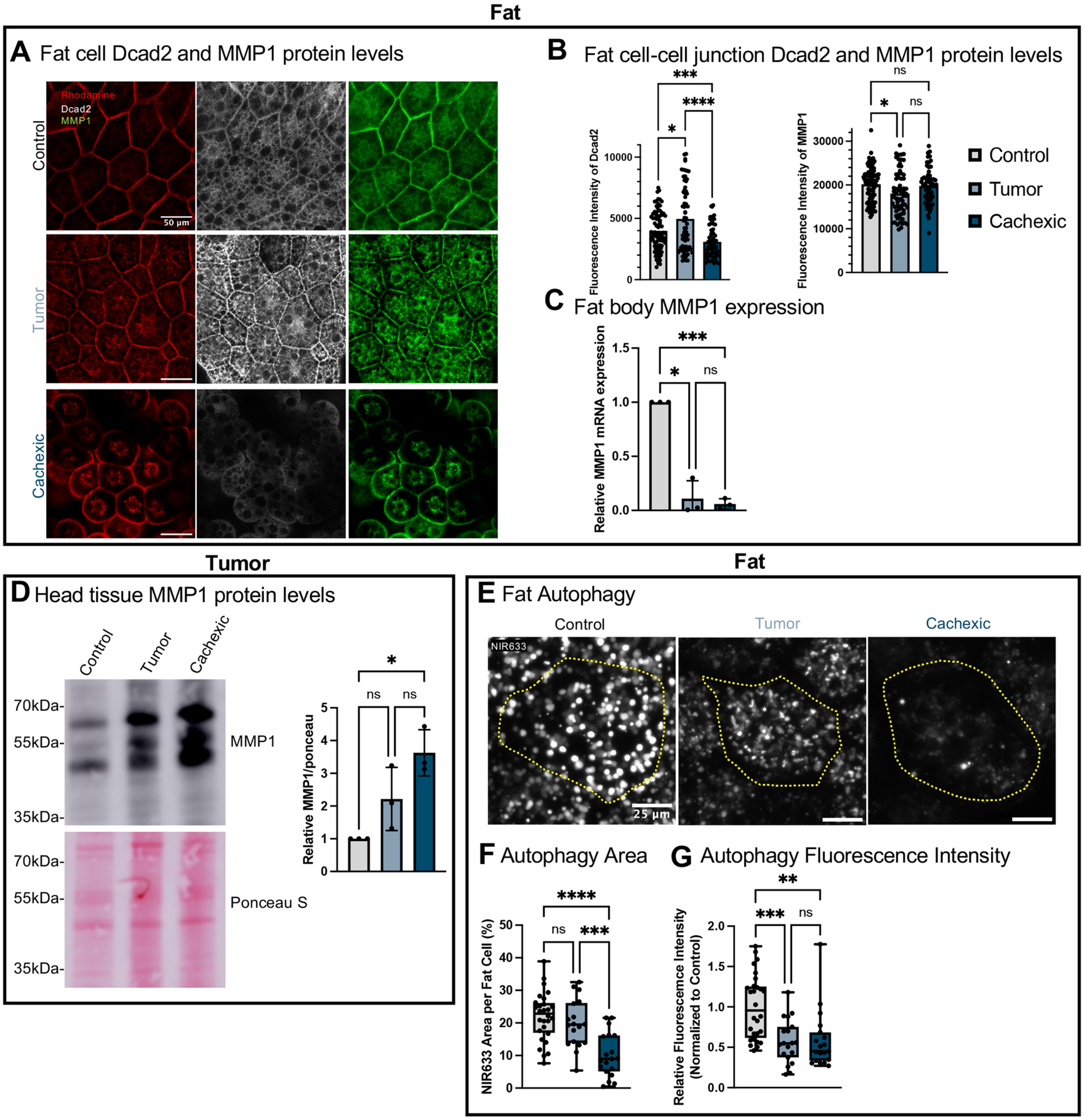

Disease Models & Mechanisms • Supplementary information

Fig. S2. Tumor potentially secretes MMP1 to reduce E-cadherin levels around fat cells causing rounding of fat cells.

(A) Single slice confocal images of F-actin staining (red), Dcad2 (white) and MMP1 (green). (n=27, 21 and 15)

(B) Quantification of fluorescence intensity of fat cell-cell junction Dcad2 and MMP1 levels. (n=89, 70 and 58) ns=0.9172 and 0.1061 respectively, *p = 0.0225, ***p = 0.0007 and ****p < 0.0001 (Brown-Forsythe and Welch ANOVA test)

(C) Fat body MMP1 mRNA levels measured by qPCR (n=5 larvae/data point) *n* = 3 biologically independent experiments ns=0.7735, *p = 0.0205 and ***p = 0.0006 (ANOVA Tukey's multiple comparisons test).

(D) Western blot of whole protein extracts of head tissue (including brain, epithelial discs and salivary glands) from third instar larvae and probing for the presence of MMP1 protein. Ponceau S is used as a loading control. Ratio of MMP1 protein level to ponceau S. ns=0.2768 and 0.3485 respectively, and *p = 0.0418 (ANOVA Tukey's multiple comparisons test).

(E) Maximum projection images of fat body stained for NIR633 (white) with fat cells outlined in yellow dotted line.

(F) Quantification of NIR633 area per fat cell. (n=28, 18 and 19) ns=0.6896, ***p = 0.0006 and ****p < 0.0001 (Brown-Forsythe and Welch ANOVA test)

(G) Quantification of NIR633 fluorescence intensity normalized to control. (n=28, 18 and 19) ns=0.9994, **p = 0.0016 and ***p = 0.0002 (Brown-Forsythe and Welch ANOVA test)

Disease Models & Mechanisms • Supplementary information

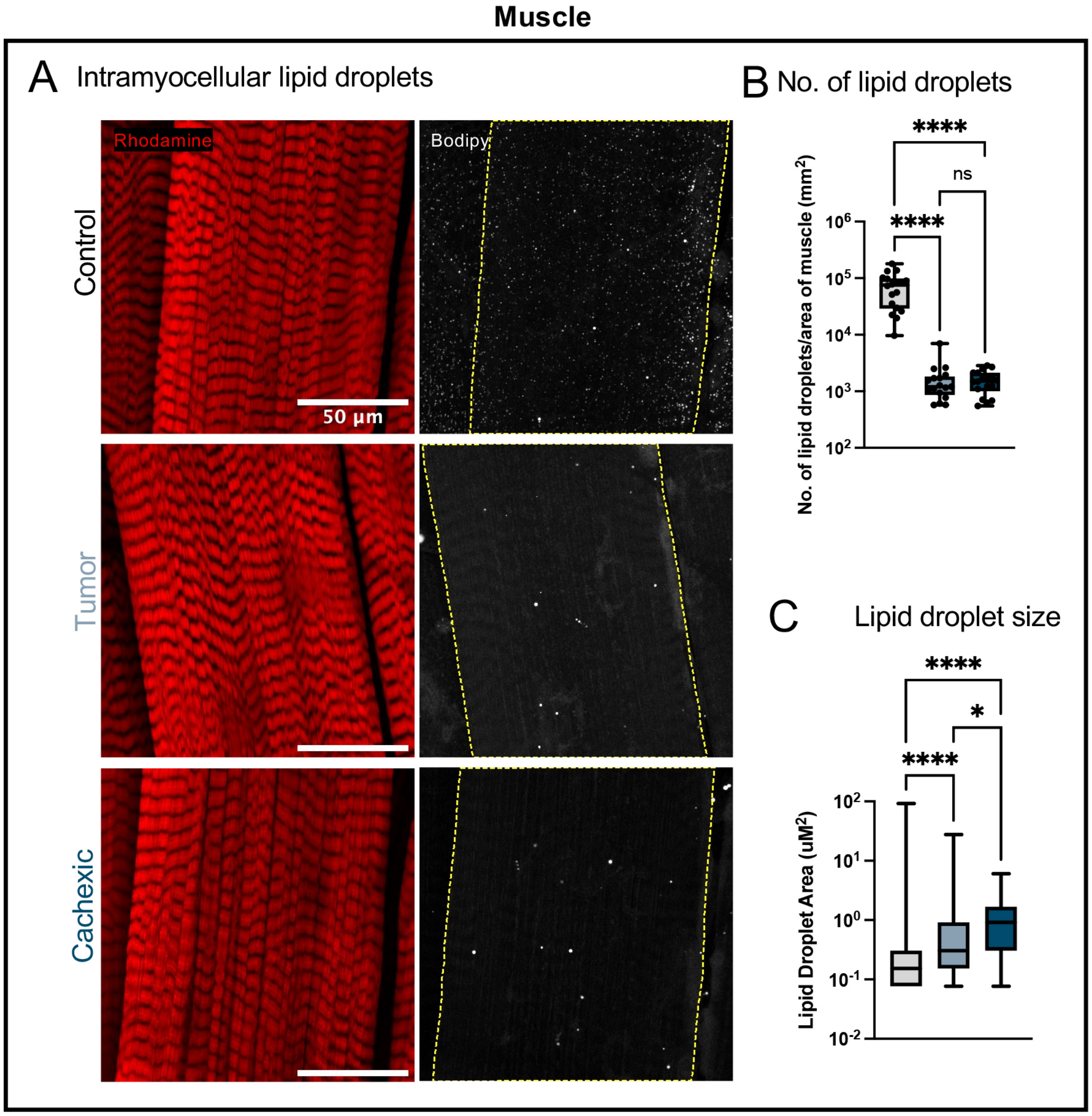

Fig. S3. Intramyocellular lipid droplets are significantly reduced in number and increased in size in tumor-bearing and cachexic larvae.

(A) Representative longitudinal maximum projection confocal images of ventral longitudinal VL4 from A2-A4 hemisegments. F-actin (red) and Bodipy (white).

(B) Quantification of number of lipid droplet within in each muscle. (n=18, 17 and 18 respectively) ****$p < 0.0001$ (Brown-Forsythe and Welch ANOVA test).

(C) Quantification of area of lipid droplet within in each muscle. (n=14962, 395 and 417 respectively) *$p = 0.0263$ and ****$p < 0.0001$ (Brown-Forsythe and Welch ANOVA test).

Disease Models & Mechanisms • Supplementary information

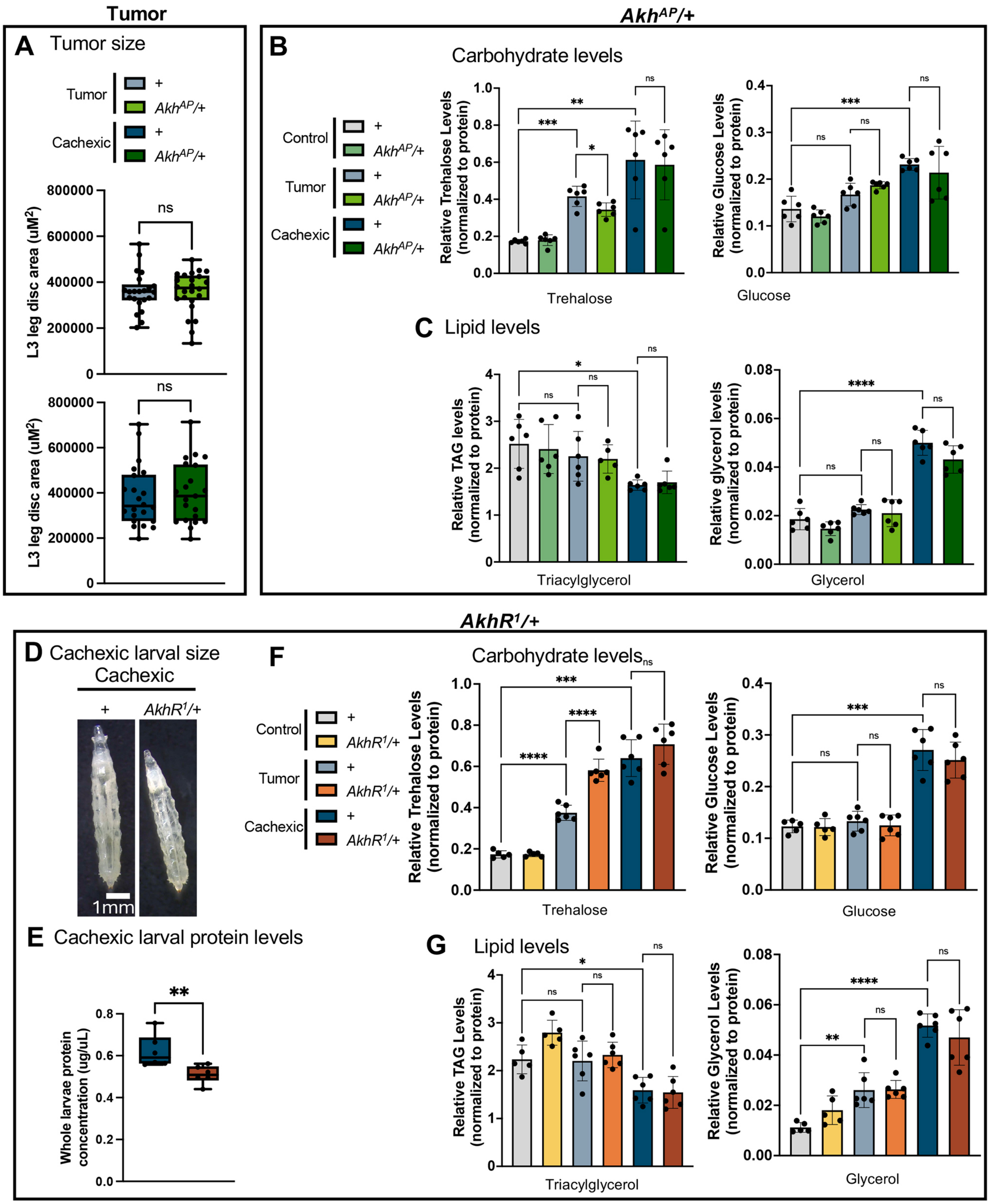

Disease Models & Mechanisms • Supplementary information

Fig. S4. Whole larval partial depletion of Akh can significantly reduce increased trehalose in tumor-bearing larvae but is unable to rescue increased lipolysis and trehalose in cachexic larvae.

(A) Quantification of L3 leg disc area for tumor and cachexic larvae (n=22, 24, 22 and 21 respectively) ns = 0.9771 and 0.6753 respectively (Welch's t test)

(B) Trehalose levels in whole larvae normalized to protein levels. (n=5 larvae/data point) ns = 0.826, *p = 0.0266, **p = 0.007 and ***p = 0.0002 respectively (Brown-Forsythe and Welch ANOVA test and Welch's t test). Glucose levels in whole larvae normalized to protein levels. (n=5 larvae/data point) ns = 0.1212, 0.4858 and 0.1045, ***p = 0.002 respectively (Brown-Forsythe and Welch ANOVA test).

(C) Triacylglycerol levels in whole larvae normalized to protein levels. (n=5 larvae/data point) ns=0.313, 0.8305 and 0.6057, *p = 0.0186 respectively (Brown-Forsythe and Welch ANOVA test). Glycerol levels in whole larvae normalized to protein levels. (n=5 larvae/data point) ns=-.1598, 0.0523, and 0.5639, ****p < 0.0001 (Brown-Forsythe and Welch ANOVA test).

(D) Representative images of cachexic larvae (n = 10 and 11 respectively).

(E) Protein concentration in whole larvae. (n=5 larvae/data point) **p = 0.0039 (Paired t test). *n* = 6 biologically independent experiments

(F) Trehalose levels in whole larvae normalized to protein levels. (n=5 larvae/data point) ns = 0.2402, ***p = 0.0001 and ****p < 0.0001 respectively (Brown-Forsythe and Welch ANOVA test and Welch's t test). Glucose levels in whole larvae normalized to protein levels. (n=5 larvae/data point) ns = 0.5169, 0.4904 and 0.3855, ***p = 0.0003 respectively (Brown-Forsythe and Welch ANOVA test).

(G) Triacylglycerol levels in whole larvae normalized to protein levels. (n=5 larvae/data point) ns=0.9845, 0.799 and 0.5487, *p = 0.0113 respectively (Brown-Forsythe and Welch ANOVA test). Glycerol levels in whole larvae normalized to protein levels. (n=5 larvae/data point) ns=0.93, and 0.3657, **p = 0.0046, and ****p < 0.0001 (Brown-Forsythe and Welch ANOVA test).

Disease Models & Mechanisms • Supplementary information

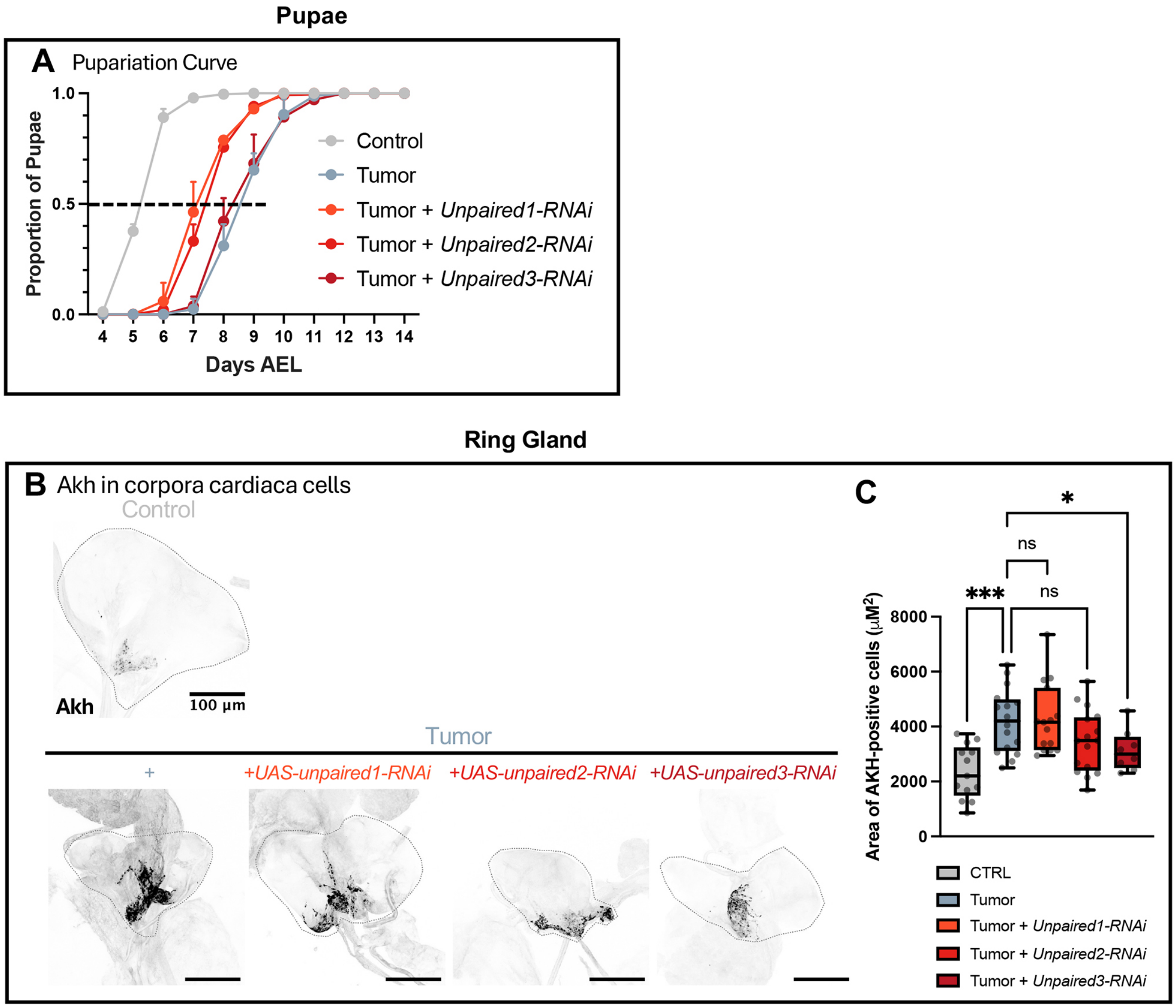

Fig. S5. Unpaired 3 ligand contributes to increased AKH levels in tumor-bearing larvae

(A) Developmental timing of larvae expressing the indicated transgenes. AEL, after egg laying. Values shown are means ± SD; *n*=15-106 pupae per biological replicate

(B) Maximal projection images of the ring gland (circled in the dotted line) of female L3 larvae immunostained for AKH (black, inverted LUT).

(C) Quantification of AKH signal area (uM^2) (n = 13, 16, 15, 16 and 8 respectively). ns=0.9996 and 0.2747 respectively and *p = 0.049 and ***p=0.003 (Brown-Forsythe and Welch ANOVA test).

Disease Models & Mechanisms • Supplementary information
